# VTA neurons coordinate with the hippocampal reactivation of spatial experience

**DOI:** 10.7554/eLife.05360

**Published:** 2015-10-14

**Authors:** Stephen N Gomperts, Fabian Kloosterman, Matthew A Wilson

**Affiliations:** 1 Department of Neurology, MassGeneral Institute for Neurodegenerative Disease, Massachusetts General Hospital, Charlestown, United States; 2 Department of Brain and Cognitive Sciences, Massachusetts Institute of Technology, Cambridge, United States; 3 Picower Institute for Learning and Memory, Massachusetts Institute of Technology, Cambridge, United States; 4 Neuro-Electronics Research Flanders (NERF), Leuven, Belgium; 5 IMEC, Leuven, Belgium; 6 Department of Psychology, Laboratory of Biological Psychology, Leuven, Belgium; Boston University, United States

**Keywords:** hippocampus, VTA, replay, dopamine, electrophysiology, sleep, Rat

## Abstract

Spatial learning requires the hippocampus, and the replay of spatial sequences during hippocampal sharp wave-ripple (SPW-R) events of quiet wakefulness and sleep is believed to play a crucial role. To test whether the coordination of VTA reward prediction error signals with these replayed spatial sequences could contribute to this process, we recorded from neuronal ensembles of the hippocampus and VTA as rats performed appetitive spatial tasks and subsequently slept. We found that many reward responsive (RR) VTA neurons coordinated with quiet wakefulness-associated hippocampal SPW-R events that replayed recent experience. In contrast, coordination between RR neurons and SPW-R events in subsequent slow wave sleep was diminished. Together, these results indicate distinct contributions of VTA reinforcement activity associated with hippocampal spatial replay to the processing of wake and SWS-associated spatial memory.

**DOI:** http://dx.doi.org/10.7554/eLife.05360.001

## eLife digest

Whether it’s meeting a friend for dinner at a new restaurant or hiking through a forest for the first time, new experiences lead to new memories. In order to create these memories, our brains do a lot of work behind the scenes. A region of the brain called the hippocampus ‘records’ experiences as they happen. It then replays these recordings later on, both while we are awake and once we have fallen asleep.

During active behaviour, dopamine cells in a different part of the brain, called the ventral tegmental area (or VTA), recognize unexpected rewards, known as “reward prediction errors”, to guide learning. For example, was the chocolate mousse or conversation unusually good, or was there an unexpected and spectacular waterfall? In order to reap rewards for our future selves, we need to remember such unexpected rewards in the context in which they occurred. However, despite the importance of this ability, research has not yet established how the brain combines our experiences as we navigate the world around us with information about reward prediction errors in our memories.

Gomperts et al. have now investigated this process by giving rats a memory task. In the first phase of the task, the rats were placed in an arena and directed to turn either left or right to receive food. In the second phase, the rats were given a choice as to whether to turn left or right. If they turned in the opposite direction to that required in the first phase, they received food rewards. The rats performed multiple trials of this task each day, and over a number of days the rats learnt the layout of the arena and the rule to locate the food. Gomperts et al. recorded the rats’ brain activity across the multiple trials of the task and after the task as the rats slept.

As expected, the findings showed that the hippocampus replayed the rats’ movements through the arena, both between the trials and after the task during sleep. Between trials, the activity of VTA neurons that encoded reward-related information coordinated with the hippocampus’ memories of paths through the arena and indicated where in the arena the rats found food rewards. Unexpectedly however, coordination of these VTA neurons with the hippocampus was greatly reduced when the rats were asleep. This observation suggests that waking memories and sleep-associated memories may serve distinct purposes, for example, to aid learning and planning or to stabilize the memories. A key challenge now is to determine these distinct roles.

**DOI:** http://dx.doi.org/10.7554/eLife.05360.002

## Introduction

Hippocampal dependent learning and memory formation are influenced by reward and are believed to occur during distinct behavioral states. As animals explore the environment, hippocampal place cells fire sequentially under the modulation of the theta rhythm. Subsequently, these sequences of neuronal activity are replayed in association with hippocampal sharp wave-ripple (SPW-R) events of quiet wakefulness and sleep (O'Keefe and Dostrovsky, 1971; Lee and Wilson, 2002; Foster and Wilson, 2006; Diba and Buzsáki, 2007). SPW-R events contribute to spatial learning (Jadhav et al., 2012; Girardeau et al., 2009; Ego-Stengel and Wilson, 2010), and the capacity for reward to influence reactivation of CA3 place cell pairs in SPW-R events (Singer and Frank, 2009) suggests that reward-related neural activity is likely to play an important role in this process. It has been unclear whether replay events of quiet wakefulness and sleep differ in their contribution to learning and memory, but the observation that replay events during slow wave sleep (SWS) are lower fidelity than replay events during quiet wakefulness (Karlsson and Frank, 2009) supports this possibility.

Dopamine neurons of the VTA represent reward prediction error Schultz, 1998 and appear to be an important brain substrate for reinforcement learning (Montague et al., 1996). Optogenetic activation of dopamine cells during spatial learning has recently been demonstrated to increase the reactivation of CA1 place cell pairs in sleep and stabilize subsequent spatial learning (Mcnamara et al., 2014). In addition, electrical stimulation of the medial forebrain bundle triggered on a hippocampal place cell’s spikes has recently been shown to drive goal-directed behavior toward its place field (de Lavilléon et al., 2015). However, it is unclear how under normal physiological conditions dopamine neuronal activity engages with the hippocampus. Dopamine cells could coordinate with and reinforce replayed hippocampal sequences. In addition, the fast-onset, slowly decaying profile of dopamine synaptic release has led to the suggestion that dopamine could implement the propagation of expected value across reactivated hippocampal sequences (Foster and Wilson, 2006). We hypothesized that replayed hippocampal spatial sequences would coordinate with reward-related representations of VTA neurons during tasks that place demands on spatial memory. Here, we acquired simultaneous multi-electrode (tetrode) recordings of neurons of the hippocampus and the VTA as rats performed appetitive spatial tasks and subsequently slept to determine the relationship between VTA neuronal activity, hippocampal SPW-R-associated activity, and sequence replay. We show that many reward responsive (RR) VTA neurons modulate their firing rate with SPW-R events of quiet wakefulness. Modulation of VTA unit activity was greater in SPW-R events associated with hippocampal replay of task-associated sequences. In contrast to nonRR VTA unit activity, RR unit activity preferentially coordinated with replayed representations of reward sites. In addition, RR VTA units more strongly phase-locked to the hippocampal theta rhythm than nonRR units, and RR VTA units that more strongly coupled to hippocampal theta had greater coordination with replayed reward site representations. In contrast to these findings in the awake state, in post-task epochs of SWS, SPW-R modulation of RR VTA unit activity was significantly reduced. Furthermore, within SWS, RR unit activity decreased during periods of hippocampal SPW-R reactivation. Together, these results indicate distinct contributions of VTA reinforcement activity associated with hippocampal spatial replay to the processing of wake and SWS-associated spatial memory.

## Results

### Coordination of VTA unit activity with hippocampal SPW-R events of quiet wakefulness

**Figure 1.**
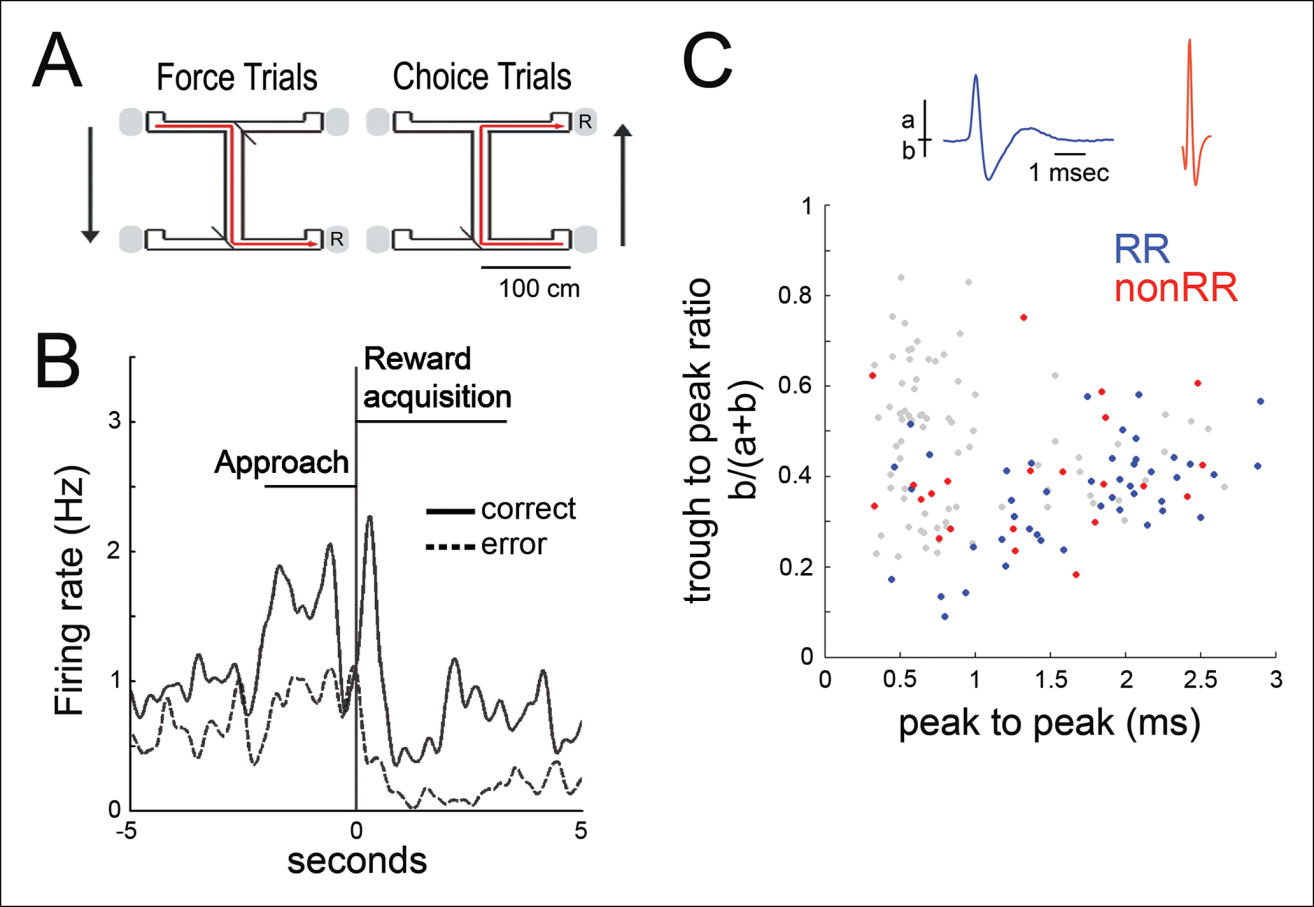
Spatial working memory task and VTA unit properties. (**A**) Spatial working memory task. In the force direction (sample phase), rats traverse the central arm for reward (**R**) at either of two pseudorandomly selected left or right force-reward locations. The reward contingency in the choice direction (test phase) required that if the rat had been forced to turn left (or right) in the sample phase, then the correct response in the test phase was to turn right (or left, respectively). (**B**) Example VTA unit’s average reward site responses for correct trials (solid line) and error trials (dashed line). The nosepoke occurs at 0 s. The profile of reward-site associated activity, including differential activity on correct versus error trials during reward approach and during reward acquisition, is consistent with prior observations in instrumental tasks (Morris et al., 2006; Roesch et al., 2007; Totah et al., 2013). (**C**) Waveform features of 145 VTA units recorded in the sleep box, using the waveform criteria described in (Fujisawa and Buzsáki, 2011). The waveform duration is defined as the time from waveform major peak to final peak. The trough to peak ratio is defined as the ratio of the waveform trough amplitude to the full amplitude. 84 units that were acquired with adequate task behavior and co-recorded hippocampal activity underwent further analysis. Reward responsive (RR) units are shown in blue, and non-reward responsive units (nonRR) are shown in red. Waveforms of two units are displayed. **DOI:** http://dx.doi.org/10.7554/eLife.05360.003

We recorded the activity of multiple simultaneously isolated units of the hippocampus (499 total; for each recording, median of 25, range 12–37) and VTA (84 total; median of 5, range 2–9) in five animals, as animals performed a spatial working memory (SWM) task (Jones and Wilson, 2005) (three rats) (Figure 1A) or ran on a linear track (two rats) for food reward. The latter task was selected both because the observation of awake replay has been best characterized in a linear environment and because it provides a choice-free spatial task for contrast. Many VTA units modified their firing rate during goal approach and with acquisition of food rewards (n = 47/84), consistent with prior observations (Morris et al., 2006; Roesch et al., 2007; Totah et al., 2013) (Figure 1B**;** Materials and methods). These results have been interpreted as the representation of reward prediction error in instrumental tasks (Morris et al., 2006; Roesch et al., 2007; Totah et al., 2013)  (specifically, the Q-associated temporal difference prediction error, where Q-value is the value of selecting a particular action at a given state). The mean firing rates for reward responsive (RR) and non-reward responsive (nonRR) VTA units were 6.61 ± 1.33 Hz (mean±s.e.m., RR units, n = 47) and 20.59 ± 5.49 Hz (nonRR units, n = 24), respectively. Two populations of cells were observed in a plot of waveform duration versus trough to peak ratio, consistent with prior reports (Fujisawa and Buzsáki, 2011) **(**Figure 1C). Most RR cells (38/47) fell in the longer duration cluster (> 1 ms), which appears to be enriched for putative dopamine cells (Fujisawa and Buzsáki, 2011; Ungless and Grace, 2012).

**Figure 2.**
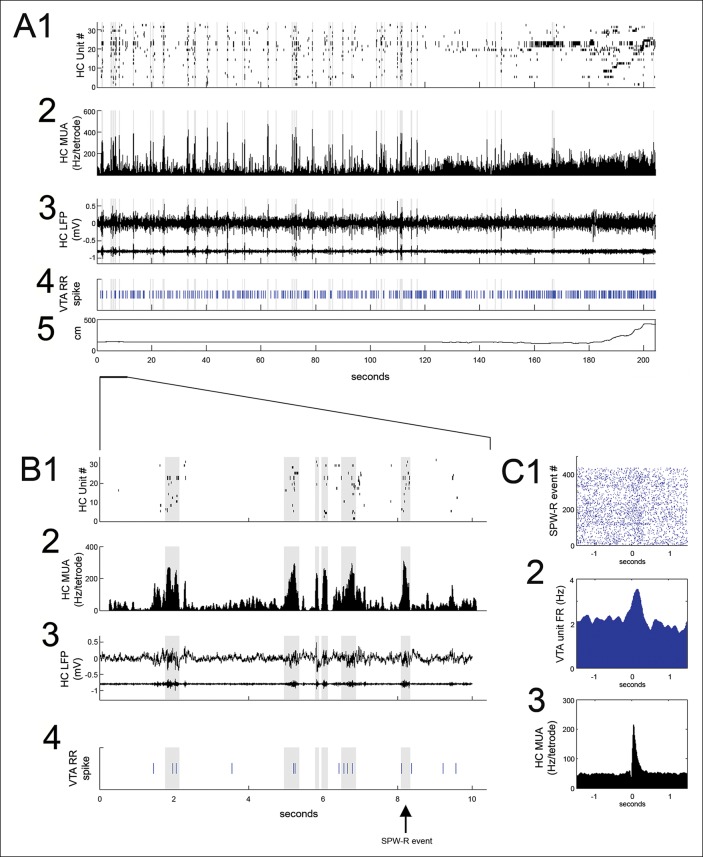
VTA unit coordination with hippocampal sharp-wave ripples. (**A**) Continuous recordings of hippocampal (HC) (**1**) single unit activity, (**2**) multiunit activity (MUA, average spike rate per tetrode), (**3**) local field potential and ripple band, (**4**) a simultaneously recorded reward-responsive (RR) VTA unit, and (**5**) the animal’s position on the track. The hippocampal units are ordered by the position of their place fields on the spatial working memory task. Sharp-wave ripple events (SPW-R) are shown in gray. (**B**) A magnified view of 10 s of continuous data. (**C1**) Rastered RR VTA unit action potentials, (**2**) RR VTA unit peri-event time histogram (PETH; smoothing with a 50ms Gaussian window), and (**3**) HC multiunit PETH (10 ms Gaussian smoothing), aligned to the start of SPW-R-associated HC multiunit events. **DOI:** http://dx.doi.org/10.7554/eLife.05360.004

SPW-R events, identified using hippocampal multiunit activity and local field potential (see Materials and methods), were prominent at reward sites during pauses in run behavior between trials (Figure 2A,B) and were measured in the period between nosepoke and run initiation to the next reward site. Reward acquisition occurred within the first 1 s of nosepoke. Reward site dwell times were variable and self-paced, with a median of 9.3 s (range: 1.5 to 615.0 s). The frequency of SPW-R events on the SWM task was higher during pauses at reward locations on correct (rewarded) trials than on error trials (correct: 0.088 ± 0.019 Hz; error: 0.029 ± 0.009 Hz; p<0.01, signed-rank test), consistent with prior results, Singer and Frank, 2009.

**Figure 2—figure supplement 1.**
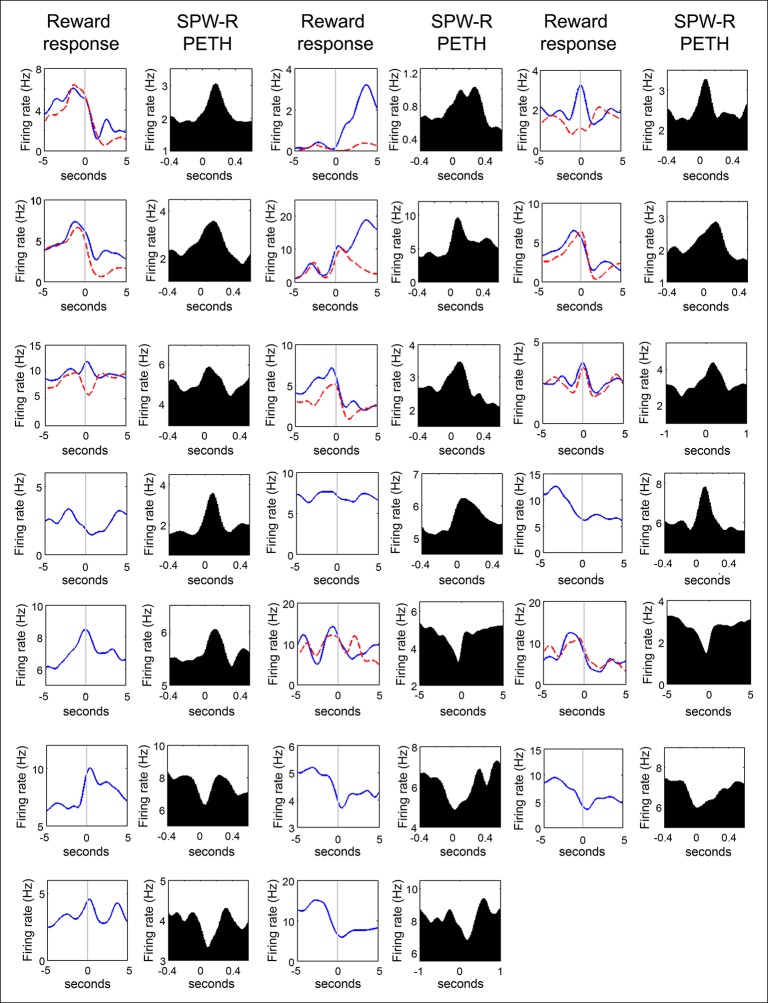
Firing rate distributions of SPW-R modulated VTA units at reward acquisition and at SPW-R events of quiet wakefulness. For units recorded on the SWM task, the average nosepoke triggered PETH for correct trials (solid blue lines) and for error trials (red dashed lines) are shown. Units acquired on the linear maze have a single nosepoke triggered PETH. Data are aligned to the time of nosepoke (vertical line). For the SPW-R event triggered PETH plots, data are aligned to the start of SPW-R events. Note that VTA unit activity often increases during reward approach and reward acquisition, and that VTA unit activity can be both positively and negatively modulated at SPW-R events. **DOI:** http://dx.doi.org/10.7554/eLife.05360.005

Many (20/84) VTA units significantly modulated their firing around SPW-R events (p<0.05, bootstrapped confidence intervals; median baseline-normalized modulation amplitude of 0.15; range 0.0003–1.41; n = 84; Figure 2). Both positive and negative SPW-R modulations were observed (positive n = 13; negative n = 7; Figure 2—figure supplement 1). Most SPW-R modulations coincided with SPW-R events; however, some negative SPW-R modulations occurred on a longer timescale, flanking SPW-R events.

The majority of VTA units that were significantly modulated at SPW-R events were RR (17/20 compared to 47/84 recorded, p=0.03, chi = 4.6, Chi-square test), and SPW-R modulation depth was greater for RR units than nonRR units (RR units 0.21 ± 0.04, n = 45; nonRR units 0.11± 0.02; p=0.017, n = 23, rank-sum test). For RR units, the sign of SPW-R modulation correlated with the sign of firing rate changes associated with reward acquisition (r = 0.55, p=1.2 x× 10^-4^). Modulation of RR units at SPW-R events did not require active reward consumption, as a similar modulation depth (0.26 ± 0.05) was noted when only SPW-R events delayed relative to nosepoke by at least 6 s were considered (signed-rank test, p=0.7). SPW-R modulation depth for RR VTA units was not significantly different on the SWM task compared to the linear track (SWM task, 0.26 ± 0.06, n = 26; linear track, 0.13 ± 0.02, n = 19, p*=*0.3, rank-sum test).

We next sought to determine whether RR unit modulation around SPW-R events was related to hippocampal replay. To evaluate spatial information associated with SPW-R replay events, we used a clusterless, probabilistic reconstruction method to maximize decoding fidelity (Kloosterman et al., 2014). First, we confirmed that the recorded hippocampal neuronal population conveyed sufficient spatial information to accurately decode the rat’s position on the track. Indeed, a cross-validation procedure showed that decoded hippocampal activity accurately reflected the rat’s location during run behavior (speed > 10 cm/s), with median error of 8.3 ± 0.5 cm across recording sessions **(**Figure 3—figure supplement 1A–C; see Materials and methods). We also confirmed that this clusterless reconstruction method resulted in lower median error than a cluster-based approach (median error 15.2 ± 1.9 cm, p=1.22 × 10^-4^, signed-rank test, n = 14 recordings).

Reconstruction of hippocampal activity during pauses in run behavior (speed < 10 cm/s; in 25 ms time bins) identified putative replay events: the representation of a sequence of locations during SPW-R events (Figure 3—figure supplement 1D). For each event, we computed the statistical likelihood that the decoded positions represented a constant-speed traversal of a trajectory on the track (Davidson et al., 2009; Kloosterman, 2012) and compared it to distributions obtained after two separate randomization procedures (see Materials and methods). Replay events identified with this approach constituted 24.8 ± 2% (1107/4645) of SPW-R events.

**Figure 3.**
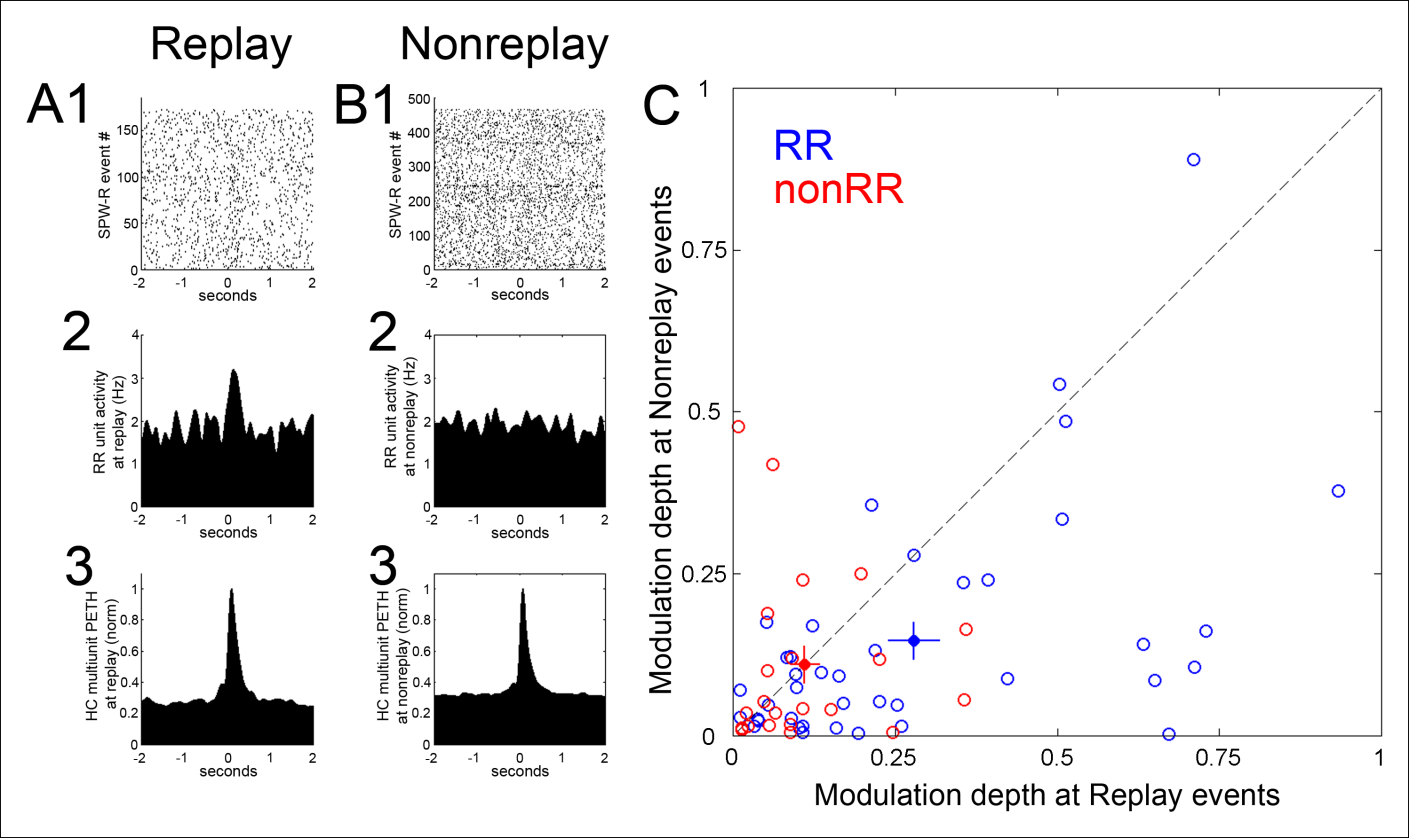
Modulation depth of VTA reward responsive units at hippocampal SPW-R events depends on SPW-R spatial content. (**A**) Rastered reward responsive (RR) unit spikes (**1**) and RR unit and hippocampal (HC) multiunit PETHs (**2,3**), aligned to the start of SPW-R events encoding replay sequences. (**B**) As in **A**, for SPW-R events not encoding replay. (**C**) PETH modulation depth of RR units (blue) is greater for replay than nonreplay events; p=4.5 × 10^-4^, signed-rank test). NonRR unit data are shown in red (p*=*0.6). Solid circles with error bars designate the mean and s.e.m. for RR and nonRR units. **DOI:** http://dx.doi.org/10.7554/eLife.05360.006

Modulation of RR VTA unit activity was greater in SPW-R events associated with replay of sequential experience of the task than in SPW-R events that were not (modulation depth 0.28 ± 0.04 vs. 0.15 ± 0.03, p=4.5 × 10^-4^, signed-rank test; n = 40; Figure 3). In contrast, for nonRR VTA units, modulation depth was similar across replay and nonreplay events (replay 0.11 ± 0.02; nonreplay 0.11 ± 0.03; p*=*0.6; signed-rank test; n = 22).

**Figure 3—figure supplement 1.**
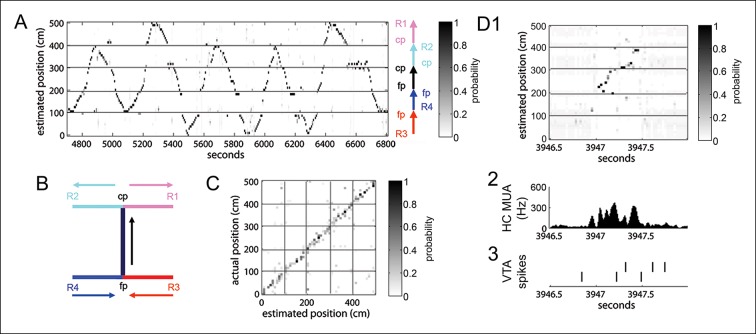
Position reconstruction using clusterless hippocampal decoding. (**A**) Bayesian reconstruction of run behavior on the spatial working memory task (500 ms time bins). The track has been linearized. (**B**) Decomposition of the track into segments for linearization. Maze segments were apposed in the direction of run in the choice direction: from force reward sites (R3, R4) to force point (fp) to choice point (cp) to choice reward sites (R1, R2). (**C**) Confusion plot for this recording session, using alternating 1 s epochs for training and testing the reconstruction algorithm. (**D1**) Bayesian reconstruction of a SPW-R event reveals spatial sequence reactivation (25 ms time bins). (**D2**) The associated hippocampal multiunit activity. (**D3**) The action potentials of two simultaneously recorded reward responsive VTA units. **DOI:** http://dx.doi.org/10.7554/eLife.05360.007

**Figure 3—figure supplement 2.**
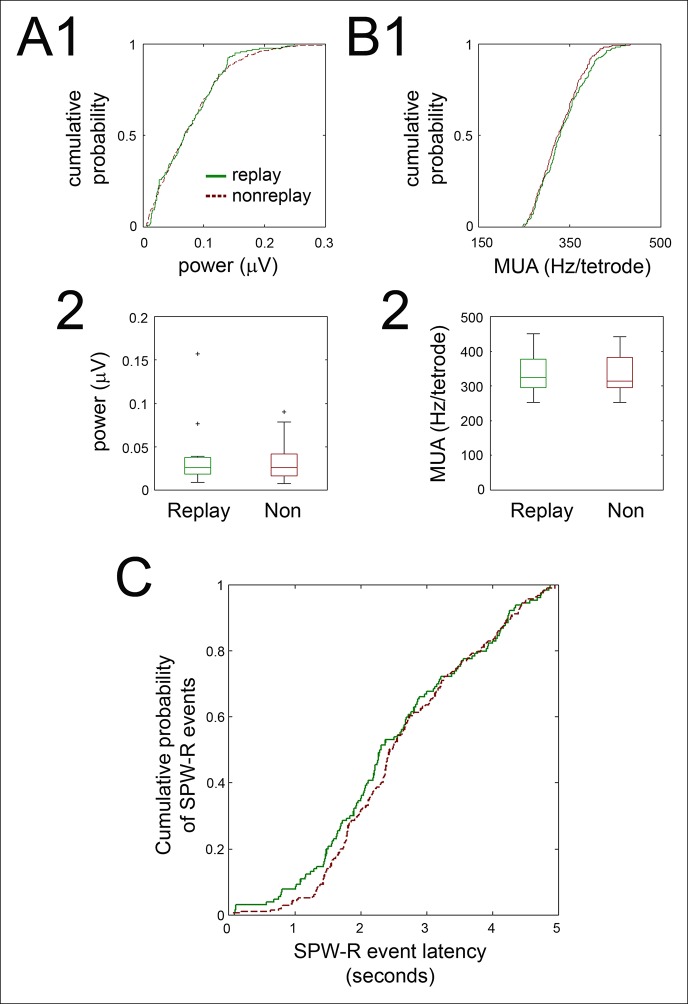
Ripple power, SPW-R associated hippocampal activity, and SPW-R event latency in the immediate post-reward period were similar for replay and non-replay events. (**A1**) For the recording shown in Figure 3A,B, cumulative ripple-band power of replay (green solid line) and non-replay (brown dashed line) events are displayed. (**A2**) Across recordings, replay and nonreplay events have similar ripple power (box and whisker plots, medians with interquartile range; p=1, sign-rank test). (**B1**) Cumulative SPW-R event peak multiunit activity (MUA; Hz/tetrode) for the same example, for replay (green solid) and non-replay (brown dashed) events. (**B2**) Across recordings, SPW-R event peak MUA is similar for replay and non-replay events (medians with interquartile range; p=0.6, sign-rank test). (**C**) Cumulative distributions of SPW-R event latencies relative to nosepoke for reward delivery were similar for replay (green solid line) and nonreplay (brown dashed line) SPW-R events (p*=*0.2, Kolmogorov-Smirnov test). **DOI:** http://dx.doi.org/10.7554/eLife.05360.008

We sought to determine whether the greater modulation of RR units around replay-associated SPW-R events compared to nonreplay-associated SPW-R events derived from some difference other than sequence replay. Ripple power and peak hippocampal firing rate at replay and non-replay SPW-R events were not significantly different (p=0.6, p=1, respectively, signed-rank tests; Figure 3—figure supplement 2A,B). Replay events had longer durations than non-replay events (0.209 ± 0.003 s vs. 0.161 ± 0.002 s, p<5.0 × 10^-16^, rank-sum test). To address whether SPW-R event duration alone could drive modulation of RR VTA unit activity, we constructed a dataset of replay and nonreplay events matched on their range of durations. Across these matched groups, the greater modulation of RR units at replay events compared to nonreplay events was preserved (replay 0.33 ± 0.05; nonreplay 0.24 ± 0.05, p=0.01, signed-rank test). Similarly, RR units at non-replay events separated by median split into short (100.6 ± 0.0 ms) and long (222.6 ± 0.1 ms) events had similar degrees of modulation (modulation depth of short events 0.20±0.04; long events 0.17 ± 0.03; p=0.9, signed-rank test; n = 40 RR units); and RR units at replay events split on median duration also did not differ in their modulation depth (short events 0.29 ± 0.04; long events 0.39 ± 0.06; p*=*0.2, signed-rank test).

Despite these similarities, replay events and nonreplay events differed with respect to the fraction of isolated pyramidal units active during each SPW-R event (replay 35.6 ± 0.0%; nonreplay 28.7 ± 0.0%, p*=*0.001, signed-rank test; n = 14 recordings) and with respect to the number of single unit action potentials per unit present in each burst (replay 1.06 ± 0.07; nonreplay 0.72 ± 0.09; p*=*6.1 × 10^-4^, signed-rank test). To address whether the greater activation of RR units around the time of replay events could be due to the greater intensity of hippocampal pyramidal cell spiking seen during these events, we constructed a dataset of spike count matched replay and non-replay events. Across the spike count matched groups, the greater modulation of RR units at replay events compared to nonreplay events was maintained (replay 0.28 ± 0.04; nonreplay 0.19 ± 0.03, p=0.03, sign rank test). Thus, the greater modulation depth of RR units at replay events compared to nonreplay events did not derive from differences in the intensity of hippocampal spiking.

We next evaluated whether the difference in RR unit coordination with replay and nonreplay events arose from differences in the timing of these events in the immediate post-reward period, when the activity of RR units often changes. Replay and nonreplay events occurring within a 5 s window from the nosepoke had similar onset latencies (replay events 2.54 ± 0.10 s, n = 130; nonreplay events 2.67 ± 0.07 s, n = 217, p*=*0.3, rank-sum test), and the temporal distributions of replay and nonreplay events following reward delivery were similar (p*=*0.2, Kolmogorov-Smirnov test; Figure 3—figure supplement 2C). These data suggest that RR units coordinate preferentially with hippocampal SPW-R events that encode within-session spatial sequences.

### Engagement of VTA unit activity with replayed spatial content

**Figure 4.**
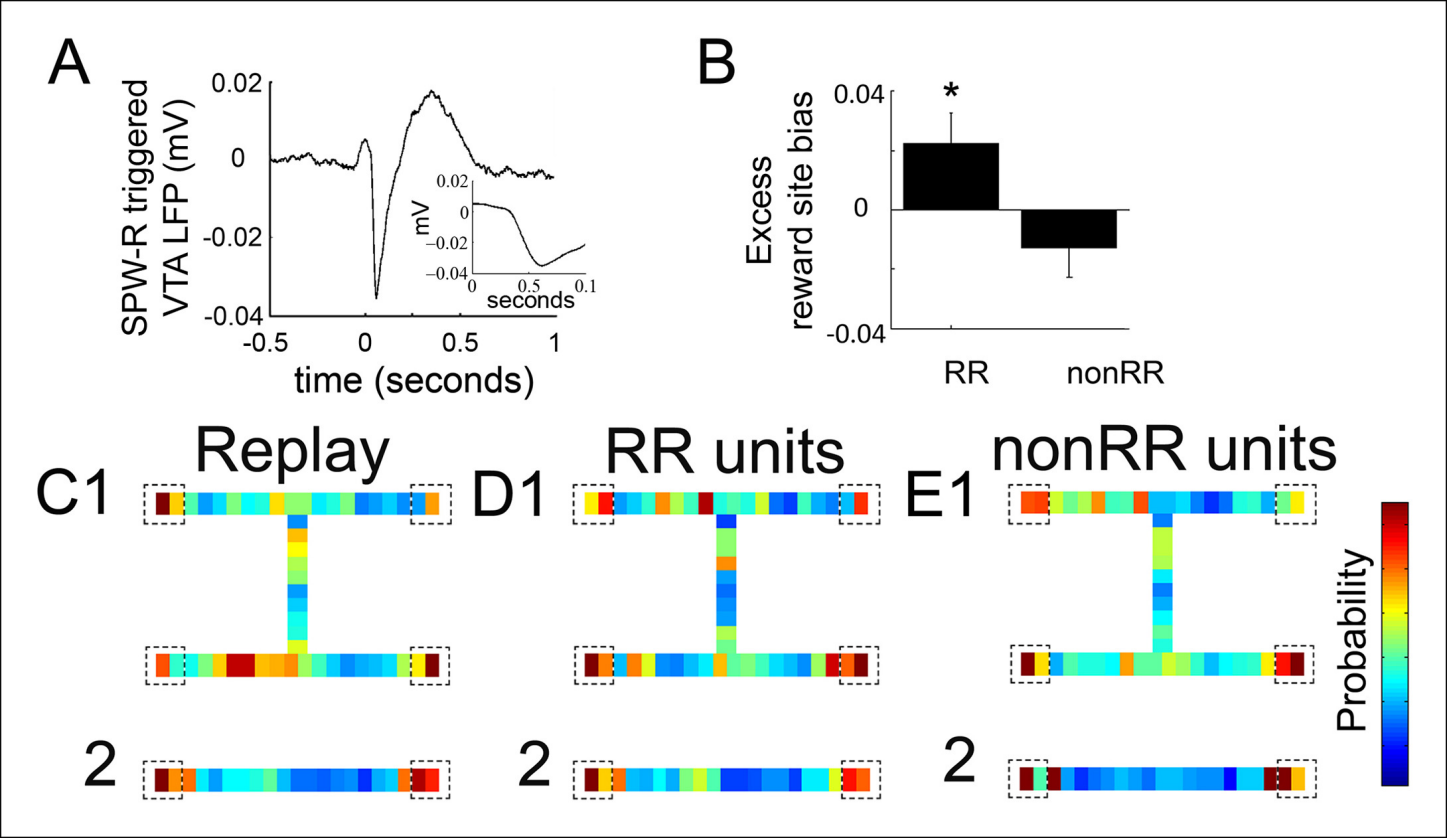
Reward responsive VTA units coordinate with replayed reward locations. (**A**) The SPW-R triggered VTA local field potential (LFP) shows a delayed potential. Time 0 reflects the start of SPW-R events. (**B**) Incorporating this delay between the hippocampus and VTA, across replay events occurring at the forced reward sites, RR unit spikes preferentially coordinated with replayed reward locations compared to SPW-R replay content in general (p=0.048, chi = 3.9, Chi-square test) and compared to nonRR units (p=0.016, nonparametric permutation test). Error bars represent s.d. (**C**) Probability distribution of replayed spatial locations for replay events occurring at the forced reward sites on the spatial working memory (SWM) (**1**) and linear tasks (**2**) (10 cm bins), accumulated across recordings. Dashed boxes designate reward sites. (**D,E**) Distribution of replayed locations coinciding with RR unit spikes (**D1,2**) and nonRR unit spikes (**E1,2**) adjusting for the latency between SPW-R onset and the VTA delayed potential. The probability colorbar for the SWM task ranges from 0 to 0.04 and for the linear track ranges from 0 to 0.1. **DOI:** http://dx.doi.org/10.7554/eLife.05360.009

The coordination of a reward prediction error signal with a hippocampal replay sequence (for example, one that represents a trajectory towards a reward) could function to reinforce specific elements of the reactivated sequence, such as a goal location. We therefore explored how VTA unit activity relates to the specific spatial locations contained in replay content. To account for latency between hippocampal SPW-R events and VTA activity, we first examined the hippocampal SPW-R event-triggered VTA LFP. This revealed a prominent negative potential that peaked 84 ± 13 ms after SPW-R onset (Figure 4A). We focused on replay events occurring at forced reward locations, which represent the beginning of choice trials and which comprised the majority of replay events (703/876, 80.3%; compared to 173 at choice reward locations). For each recording, we constructed a distribution of all decoded locations within these replay events and compared this to the distribution of decoded spatial locations specifically associated with 84 ms delayed RR VTA unit spikes and nonRR VTA unit spikes.

Across replay events, the probability of decoded spatial locations was biased toward reward sites (probability/spatial bin of replay content at reward locations 0.038 ± 0.005 bin^-1^; non-reward locations 0.021 ± 0.002 bin^-1^; p=2.4 × 10^-4^, signed-rank test; n = 14 recording sessions; see Materials and methods; Figure 4C). Incorporating the latency of 84 ms, the timing of RR VTA unit activity within replay events specifically coincided with the replay of reward locations (probability/spatial bin of VTA unit activity at reward locations 0.044 ± 0.003 bin^-1^; non-reward locations 0.021 ± 0.001 bin^-1^; p=9.0 × 10^-8^ signed-rank test; n = 41 RR units). Across all recordings, RR VTA unit spikes were biased to coincide with replayed reward site locations in excess of the proportion of reward site locations within replay events (reward site bias for replay events: 0.451±0.007; excess reward site bias for RR VTA units: 0.022 ± 0.011 (mean ± s.d.); p=0.048, chi = 3.9, Chi-square test; see Materials and methods; Figure 4B,D). In contrast, nonRR units did not preferentially coordinate with replayed reward site locations (excess reward site bias for nonRR VTA units: -0.013 ± 0.010; p=0.3, chi = 1.2, Chi-square test; Figure 4B,E). The contrast of excess reward site bias of RR units and nonRR units was significant (p<0.016, nonparametric permutation test; see Materials and methods). The bias in coordination of RR units but not nonRR units with replayed reward locations persisted when the current location of the animal was excluded from the analysis (reward site bias for replay events: 0.331 ± 0.009; excess reward site bias for RR VTA units: 0.031 ± 0.014; p=0.041 chi = 4.2 Chi-square test; nonRR VTA units: 0.008 ± 0.013; p=0.6, chi = 0.2, Chi-square test). These data suggest that RR VTA units preferentially associate with the hippocampal replayed representation of reward locations.

To evaluate the task dependence of this observation, we compared the preferential coordination of RR units with replayed reward site locations on the SWM task and on the linear track. Interestingly, the excess reward site bias of RR units was greater on the SWM task than on the linear track (excess reward site bias for RR units on the SWM task 0.027 ± 0.014; excess reward site bias for RR units on the linear track 0.017 ± 0.015; p=0.045, nonparametric permutation test). Because this task-dependence could reflect a role for the coordination of RR units with replayed reward locations in choice behavior, we examined whether the preferential coordination of RR units with replayed reward site locations reflected recent choice behavior or predicted future choice behavior on the SWM task. However, we were unable to detect a difference in the excess reward site bias of RR units at replay events immediately following correct trials versus error trials (excess reward site bias after correct trials 0.022 ± 0.020; after error trials -0.018 ± 0.031; p=0.19, nonparametric permutation test). Similarly, the excess reward site bias of RR units at replay events was no greater immediately prior to correct trials than prior to error trials (excess reward site bias prior to correct trials 0.001 ± 0.021; prior to error trials 0.013 ± 0.041, p=0.6, nonparametric permutation test). These results suggest that the preferential coordination of RR unit activity with replayed reward locations is task dependent but may not simply recapitulate or predict immediate reward-associated experience.

### Coordination of VTA units with specific replayed spatial sequences

**Figure 5.**
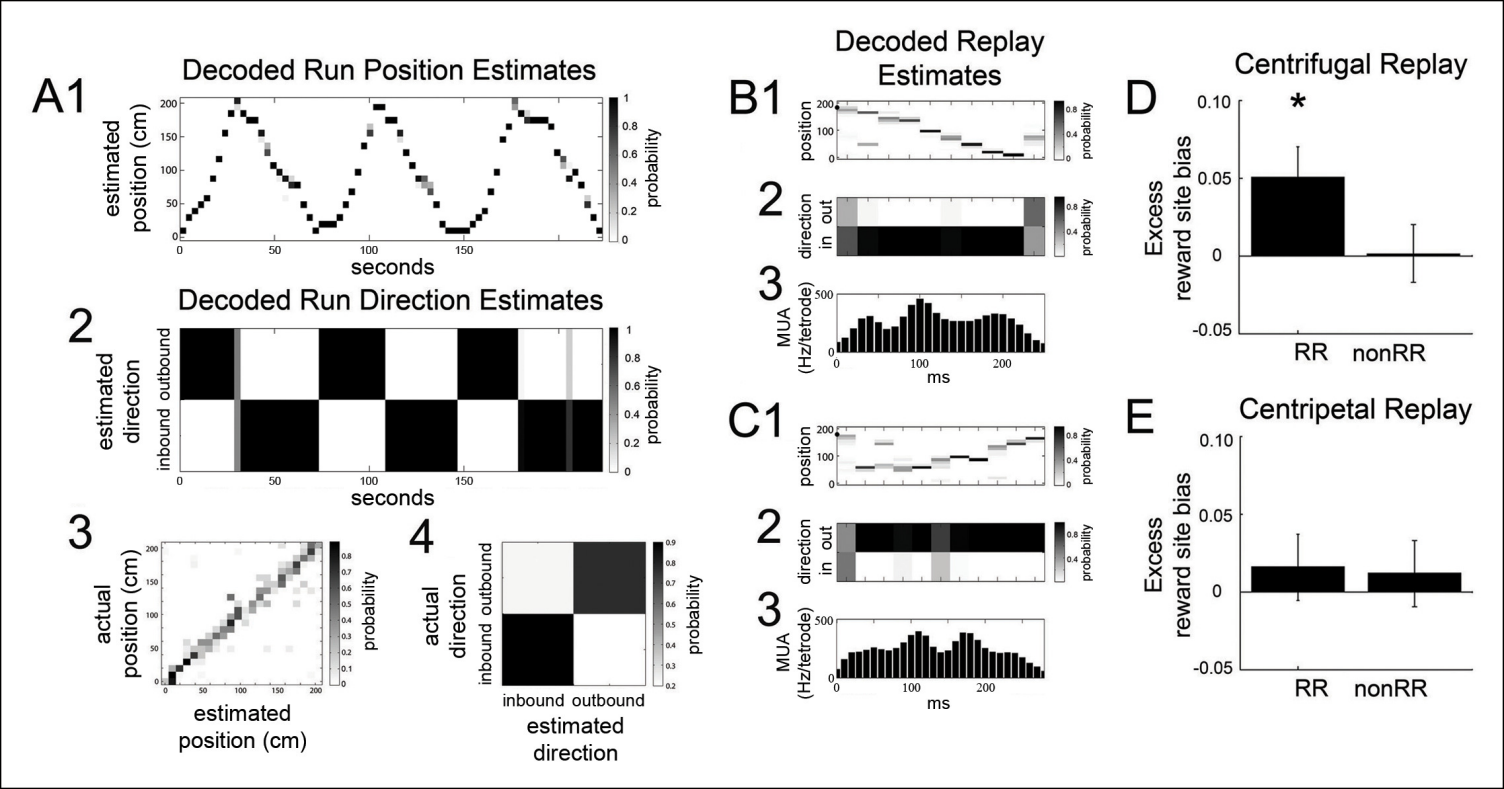
The bias of reward responsive VTA unit activity towards the replay of reward locations is greater for centrifugal than centripetal replay. (**A1,2**) Bayesian reconstruction of run position and run direction on the linear track (500 ms time bins). Outbound refers to run direction from 0 to 200 cm. (**A3**) Position confusion plot for this recording session, using alternating 1 s epochs for training and testing the reconstruction algorithm. (**A4**) Run direction confusion plot. (**B**) Centrifugal, forward replay event occurring while the rat paused at the far reward site (190 cm; black circle indicates the rat’s position). (**B1**) Position reconstruction (25 ms time bins). (**B2**) direction reconstruction). (**B3**) The associated hippocampal multiunit activity. (**C**) Centripetal, forward replay event occurring while the rat paused at the far reward site. (**C1**) Position reconstruction. (**C2**) Direction reconstruction. (**C3**) Associated hippocampal multiunit activity. (**D**) Across centrifugal replay time bins, RR unit spikes preferentially coordinated with replay of reward locations compared to centrifugal replay content in general (p=0.014, chi=6.0, Chi-square test) and compared to nonRR units at centrifugal replay (p=0.05, nonparametric permutation test). Error bars represent s.d. (**E**) Across centripetal replay time bins, RR unit spikes showed no increase in coordination with replay of reward locations compared to centripetal replay content in general (p=0.5, chi=0.5, Chi-square test). Error bars represent s.d. **DOI:** http://dx.doi.org/10.7554/eLife.05360.010

Previous work has posited a specific coordination between dopamine neuronal activity and replay events comprised of spatial sequences starting locally and replaying away from the animal (centrifugal events) in reverse order compared to their order during behavior (Foster and Wilson, 2006). To address whether the preferential coordination of RR VTA units with replayed reward site locations may derive from a selective engagement of VTA units with centrifugal replay events or with reverse replay events, in distinction from replay sequences that start remotely and replay towards the animal (centripetal events), or forward replay sequences that replay in the same order as they did during behavior, we first differentiated between instantaneous centrifugal and centripetal spatial content, and between forward and reverse spatial content, by reconstructing SPW-R replay events at forced reward sites using both position and run direction data (Figure 5A–C; see Materials and methods). We accumulated centrifugal, centripetal, forward, and reverse replayed spatial distributions separately, and we compared them to each other and to the distribution of decoded spatial locations and run direction specifically associated with RR VTA unit spikes.

**Figure 5—figure supplement 1.**
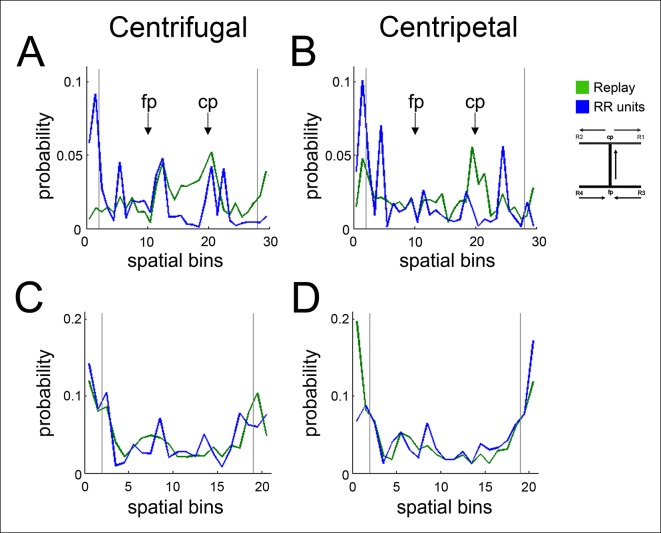
Centrifugal and centripetal replayed locations associated with RR unit activity on the SWM task and the linear track. (**A**) On the SWM task, the distribution of centrifugal replayed locations (green) is less concentrated at reward sites (marked by vertical lines) than the distribution of RR unit-associated centrifugal replayed locations (blue). See Figure 5 for statistics. Maze segments were aggregated by apposing them in the direction of run in the choice direction: from force reward sites (R3, R4) to force point (fp) to choice point (cp) to choice reward sites (R1, R2). Spatial bins 1–10 show the average of the force arms of the task (arms 3 and 4), spatial bins 11–20 show the central arm, and spatial bins 21–30 show the average of the choice arms (arms 1 and 2). (**B**) The distribution of centripetal replayed locations (green) on the SWM task is similar to the distribution of RR unit-associated centripetal replayed locations (blue). (**C**) On the linear track, the distributions of centrifugal replayed locations (green) and RR unit-associated centrifugal replayed locations (blue) are similar. (**D**) On the linear track, the distribution of centripetal replayed locations (green) and the distribution of RR unit-associated centripetal replayed locations (blue) are also similar. **DOI:** http://dx.doi.org/10.7554/eLife.05360.011

RR units preferentially coordinated with the reward site representation of centrifugal replay events but not centripetal replay events (reward site bias for centrifugal replay spatial content 0.493 ± 0.005; excess reward site bias for RR units at centrifugal replay 0.051 ± 0.020; p=0.014, chi = 6.0, Chi-square test; reward site bias for centripetal replay spatial content 0.524 ± 0.006; excess reward site bias for RR units at centripetal replay 0.016 ± 0.021; p=0.5, chi = 0.5, Chi-square test; Figure 5D,E). In contrast, nonRR units showed no excess reward site bias for replayed spatial content (excess reward site bias for nonRR units at centrifugal replay 0.002 ± 0.019, p=1, chi = 0.001, Chi-square test; at centripetal replay: 0.011 ± 0.022; p=0.6, chi = 0.2). These results demonstrate that RR units preferentially coordinate with centrifugal replay content.

We next examined RR and nonRR unit activity associated with forward and reverse replay events. The probability of decoding spatial locations at reward sites was similar for forward and reverse replay events (reward site bias for forward replay spatial content 0.422 ±  0.013; reward site bias for reverse replay spatial content 0.419 ± 0.014; p=0.9, chi = 0.2, Chi-square test). We did not detect a selective engagement of RR units with reward locations of reverse replay over forward replay (excess reward site bias for RR units at reverse replay 0.035 ± 0.020; at forward replay 0.034±0.020; p=1, nonparametric permutation test).

To evaluate the task dependence of the preferential coordination of VTA RR units with centrifugal replay spatial content, we analyzed the SWM task and the linear track separately. Similar to the results described above, RR units acquired on the SWM task preferentially coordinated with reward site representations of centrifugal replay events (excess reward site bias for RR units at centrifugal replay: 0.071 ± 0.032; p=0.028, chi = 4.8) in contrast to centripetal replay events (excess reward site bias for RR units at centripetal replay: 0.031 ± 0.028 p=0.3, chi = 1.0; Figure 5—figure supplement 1). However, this preferential coordination was not observed on the linear track (excess reward site bias for RR units at centrifugal replay on the linear track: 0.040 ± 0.024; p=0.11, chi = 2.5; excess reward site bias for RR units at centripetal replay: -0.004 ± 0.031; p=0.9, chi = 0.01). Thus, the coordination of VTA RR units with centrifugal replay of reward site locations was stronger on the SWM task.

### Phase-locking of VTA units to hippocampal theta during run behavior correlates with the engagement of VTA units with replayed spatial content

**Figure 6.**
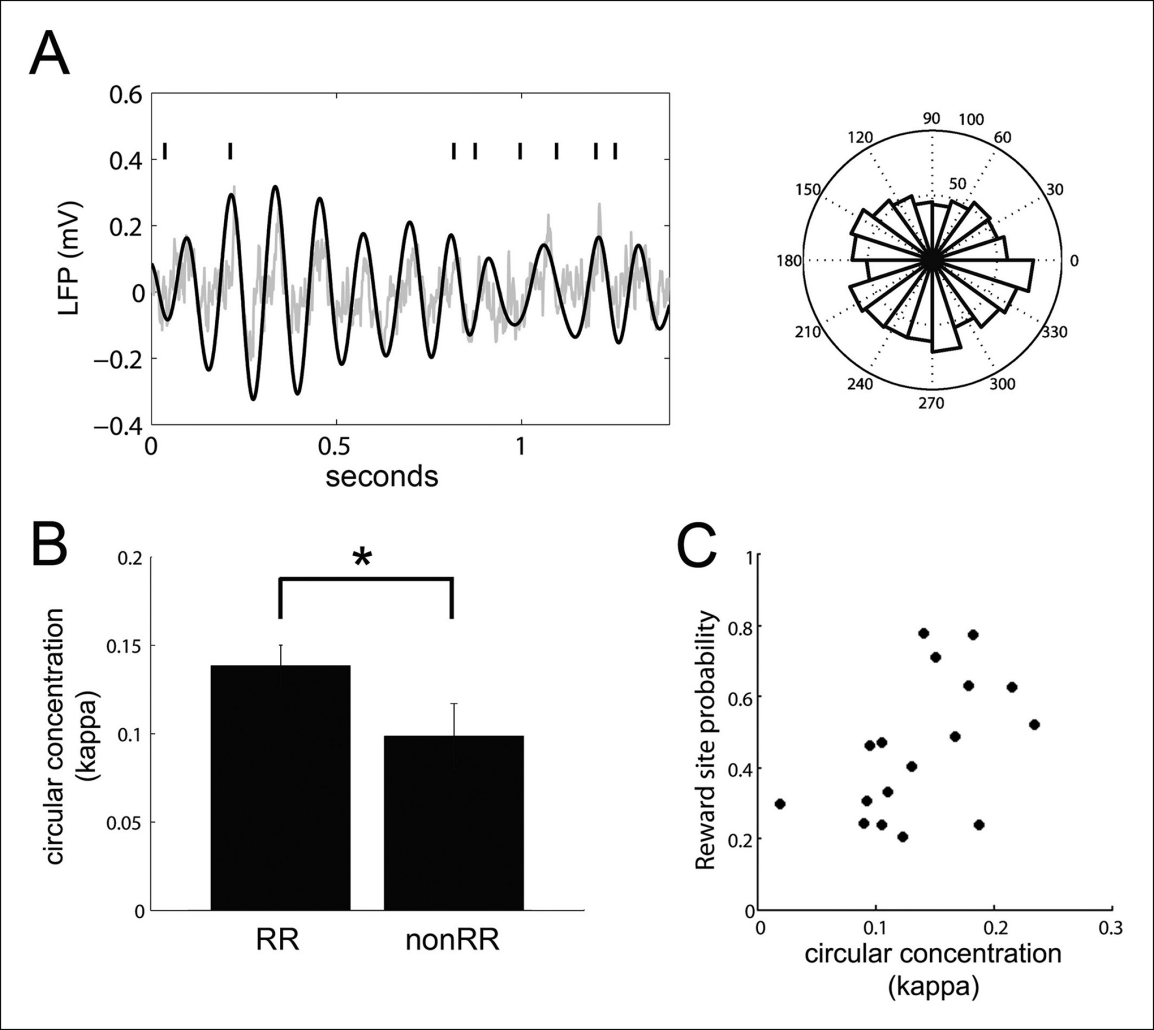
VTA units coordinate with hippocampal theta. (**A**) Spike times of a reward responsive (RR) VTA unit relative to hippocampal theta and raw LFP during running behavior, and spike phase distribution (circular concentration coefficient, kappa = 0.14; Rayleigh statistic p value = 0.002). (**B**) Circular concentration at hippocampal theta is greater for RR units than nonRR units (p=0.031, rank-sum test). Error bars represent s.e.m. (**C**) The probability of replayed reward locations coinciding with the spikes of theta-modulated RR units correlates with the circular concentration of those units at hippocampal theta (r = 0.51, p*=*0.038). **DOI:** http://dx.doi.org/10.7554/eLife.05360.012

In addition to modulating their activity at hippocampal SPW-R events, many VTA units (39/84; 43%) phase-locked to hippocampal theta during run behavior (Rayleigh test for uniformity against unimodal alternative p<0.05, phase preference -10 ± 16 degrees, relative to the peak of theta), consistent with previous observations (Fujisawa and Buzsáki, 2011) **(**Figure 6). The coordination of neural activity with the hippocampal theta rhythm has been proposed to be a mechanism used in spatial working memory (Jones and Wilson, 2005; Fujisawa and Buzsáki, 2011). We therefore sought to determine the extent to which theta phase-locking of VTA units predicted their coordination with SPW-R events. Circular concentration of VTA unit spikes around the mean preferred hippocampal theta phase was greater for RR units than nonRR units, as measured with the circular concentration coefficient kappa as described previously (Jones and Wilson, 2005; Siapas et al., 2005), (mean ± s.e.m., RR: 0.139 ± 0.012, n = 47; nonRR: 0.099 ± 0.019, n = 24; p*=*0.03, rank-sum test; Figure 6B). For theta-modulated RR units but not theta-modulated nonRR units, circular concentration at hippocampal theta positively correlated with the probability that spike-associated replayed spatial content represented reward locations (RR units: r = 0.51, p*=*0.04, n = 17; nonRR units: r = 0.45, p*=*0.13, n = 13; Figure 6C). In contrast, the circular concentration coefficient of VTA units at hippocampal theta did not correlate with the firing rate of those units (RR units: r = -0.22, p*=*0.4, n = 18; nonRR units: r = -0.49, p*=*0.09, n = 13). In addition, the circular concentration of RR units at hippocampal theta did not correlate with their modulation depth at SPW-R replay events (r = 0.12, p*=*0.5, n = 45). Thus, phase-locking of RR units to hippocampal theta during run behavior was associated with the timing but not the number of spikes of RR units at SPW-R events.

### Coordination between VTA unit activity and hippocampal SPW-R events in slow wave sleep

Previous work has demonstrated hippocampal SPW-R replay during SWS (Lee and Wilson, 2002; Pavlides and Winson, 1989; Wilson and Mcnaughton, 1994; Nádasdy et al., 1999; Ji and Wilson, 2007) and has shown that medial forebrain bundle stimulation triggered on place cell activity in sleep exerts a powerful influence on post-sleep behavior (de Lavilléon et al., 2015). Having identified replay-related modulation of VTA unit activity, we therefore sought to evaluate SPW-R-associated VTA unit activity in SWS acquired immediately subsequent to run behavior.

**Figure 7.**
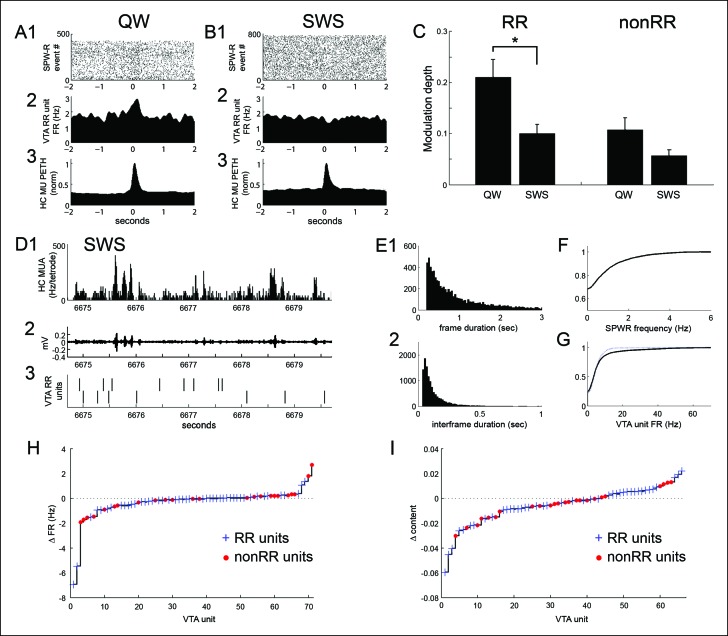
SPW-R-associated modulation of VTA units during periods of quiet wakefulness (QW) on the task and during subsequent slow wave sleep (SWS). (**A**) Rastered QW-associated reward responsive (RR) VTA unit spikes (**1**) and RR unit and hippocampal (HC) multiunit PETHs (**2,3**), aligned to SPW-R events. (**B**) SWS-associated data for the same RR unit. (**C**) SPW-R event modulation depth of RR and nonRR unit activity in QW and SWS (RR units: QW vs SWS, p=0.003, rank-sum test; nonRR units: QW vs SWS, p*=*0.13, rank-sum test). Error bars represent s.e.m. (**D1**) Hippocampal multiunit activity, (**2**) ripple band, and (**3**) two RR VTA units in SWS. (**E**) Distributions of (**1**) SWS frame duration and (**2)** interframe duration across recordings. (**F**) Cumulative distribution of within-frame SPW-R frequency. (**G**) Within-frame VTA unit activity. RR units are shown separately in dashed line. (**H**) The difference in each VTA unit’s activity at frames of high and low SPW-R rate, defined relative to the mean (RR units: p=0.003, signed-rank test; nonRR units: p*=*0.5). (**I**) The difference in mean spatial content of frames with high and low VTA unit activity, relative to the mean (RR units: p=0.045, signed-rank test). **DOI:** http://dx.doi.org/10.7554/eLife.05360.013

Both RR and nonRR VTA units reduced their firing rates in SWS (RR units: run 8.8 ± 1.7 Hz, quiet wakefulness 6.6 ± 1.3 Hz, SWS 4.5 ± 0.8 Hz; run vs SWS, p=2.2 × 10^-7^; quiet vs SWS, p=2.3 × 10^-5^; signed-rank tests, n = 47; nonRR units: run 26.8 ± 7.2 Hz, quiet wakefulness 20.6 ± 5.4 Hz, SWS 10.4 ± 2.7 Hz; run vs SWS, p=7.1 × 10^-5^; quiet vs SWS, p=1.0 × 10^-4^; signed-rank tests, n = 24). In addition, the modulation depth of RR unit activity at SPW-R events was significantly reduced in SWS (modulation depth in quiet wakefulness 0.21 ± 0.04; in SWS 0.10 ± 0.02; p=0.003, rank-sum test, n = 45 in quiet wakefulness, n = 39 in SWS; Figure 7A–C). In contrast to the awake state, RR units in SWS were often negatively modulated at SPW-R events (30/39 units, p=0.001, sign test). Modulation depths in quiet wakefulness and sleep were significantly correlated (modulation depth, r = 0.47, p=0.002, n = 39), but the sign of modulation across these states was not (modulation sign, r = 0.13, p=0.4, n = 39). In contrast to RR units, the SPW-R modulation of nonRR units was not significantly modified by behavioral state (modulation depth in quiet wakefulness 0.11 ± 0.02; SWS 0.06 ± 0.01; p*=*0.13, rank-sum test, n = 23 in quiet wakefulness, n = 20 in SWS; Figure 7C). Thus, RR units coordinated more robustly with hippocampal SPW-R events of quiet wakefulness than with those of SWS.

The smaller modulation of RR units with SPW-R events in SWS compared to quiet wakefulness could result from a difference in state or from a difference in the spatial content expressed in these states. For example, replayed spatial content in sleep may be less biased by recent experience than replayed spatial content in quiet wakefulness. To explore this question, we set out to examine hippocampal replay in detail in SWS.

We first evaluated the prevalence of replay-associated SPW-R events in SWS. Hippocampal replay of recent experience during SWS identified using Bayesian position reconstruction was less prevalent than during quiet wakefulness (SWS 16.0 ± 1.7% (800/5820), compared with 24.8 ± 2% of wake-associated SPW-R events, p=0.003, signed-rank test).

Hippocampal activity in SWS is characterized by epochs of up-state-like neuronal population activity known as frames, within which SPW-R events occur and during which coordinated replay between the hippocampus and neocortex has been observed (Ji and Wilson, 2007). Because hippocampal replay in SWS has been associated with SPW-R events within frames, we compared VTA unit activity across frames associated with high versus low SPW-R rates (SPW-R events per second), relative to the mean. RR unit firing rate was lower in high rate SPW-R frames than in low rate SPW-R frames (high rate SPW-R frames 4.23 ± 0.74 Hz; low rate SPW-R frames 4.64 ± 0.77 Hz; signed-rank test, p=0.003, n = 47; Figure 7D–H). In contrast, nonRR unit firing rate was similar across these groups of frames (high rate SPW-R frames 10.26 ± 2.66 Hz; low rate SPW-R frames 10.41 ± 2.65 Hz; signed-rank test, p=0.45, n = 24; Figure 7D–H). Thus, RR unit activity was biased away from SPW-R rich frames.

We next asked whether VTA unit firing rates varied with frame-associated replay of recent experience. For this purpose, we evaluated average spatial content in each frame, measured as the average across time bins of the maximum decoded probability at each time bin. RR unit firing rate was lower in frames associated with higher spatial content (above the mean spatial content of frames) than in frames associated with lower spatial content (below the mean; RR unit firing rate at high spatial content frames 4.11 ± 0.78 Hz vs. firing rate at low spatial content frames 4.83 ± 0.91 Hz, p*=*0.001; signed-rank test, n = 42). Frames with higher spatial content were longer than frames with lower spatial content (median 3.98 s vs 1.85 s; p*=*0.009; signed-rank test, n = 14 recordings). Because differences in frame duration could affect estimates of within-frame firing rates, we also performed an inverse analysis, in which we sorted hippocampal frames by the associated firing rate of VTA units and then compared their spatial content associated with recent experience. Frames associated with high RR unit firing rate (above the mean firing rate for each unit) had lower mean spatial content than frames associated with low (below the mean) RR unit firing rate (spatial content at high firing rate frames 0.185 ± 0.012 (a.u.) vs. spatial content at low firing rate frames 0.191 ± 0.013 (a.u.); signed-rank test, p=0.045, n = 42; Figure 7I). Together, these results demonstrate that SWS is associated with reduced SPW-R modulation of RR unit activity and with reduced RR unit activity in hippocampal frames containing a high rate of SPW-R events and in frames associated with high spatial information about recently explored environments.

## Discussion

Hippocampal dependent learning and memory are influenced by reward, and SPW-R events contribute to these functions (Jadhav et al., 2012; Girardeau et al., 2009; Ego-Stengel and Wilson, 2010). As VTA dopamine cells are driven by reward prediction errors (Schultz, 1998) and have been suggested to provide an error signal to guide learning in downstream brain regions (Montague et al., 1996), we posited that the coincidence of dopamine neuronal activity with sequence replay in SPW-R events of quiet wakefulness could reinforce spatial experience, mediating the influence of reward on hippocampal dependent processing (Singer and Frank, 2009) and memory formation.

Taken collectively, the results of this study support this possibility, demonstrating that during quiet wakefulness but not SWS, RR VTA neurons coordinate selectively with hippocampal replay sequences and are biased in their timing towards the reactivated representation of rewarded locations. In contrast, nonRR VTA neurons did not coordinate with the specific spatial content of replay sequences. RR neurons were also more strongly phase-locked to hippocampal theta than nonRR neurons, and the extent of phase-locking correlated with the coordination of RR unit activity with replayed reward locations. Previous work has demonstrated theta phase-dependent interactions between the hippocampus, prefrontal cortex, and VTA during working memory-dependent, single trial decisions (Jones and Wilson, 2005; Fujisawa and Buzsáki, 2011). Our data support a model in which these experience-dependent associations, once established, are re-expressed in SPW-Rs of quiet wakefulness, to guide spatial memory across trials. In addition, our results identify two possible endogenous substrates by which optogenetically released dopamine can increase off-line reactivation of hippocampal cells and improve spatial memory performance (Mcnamara et al., 2014): direct coordination of dopamine neuronal activity with hippocampal replay of quiet wakefulness, or coordination with hippocampal theta triggering a subsequent reactivation of dopamine neurons that engages with hippocampal replay.

The sign of SPW-R modulation varied across RR neurons, often recapitulating their reward-associated modulation of firing rate. This result suggests that as a population, RR neurons replay their reward-related activity in concert with hippocampal sequence replay, to selectively reinforce reward-associated behavior. Coordination with replay has previously been observed in neurons of the primary visual cortex (Ji and Wilson, 2007) and the striatum (Pennartz et al., 2004; Lansink et al., 2009), a major target of the VTA that represents rewards (Schultz et al., 1992; Cardinal et al., 2002). The current results extend these findings, supporting the hypothesis that replay events engage both cortical and subcortical structures to create an accurate memory trace of recent experience.

In this study, RR neurons preferentially coordinated with SPW-R events of quiet wakefulness compared to SWS and were least active in SWS frames associated with high spatial content. Although we observed a higher proportion of replay events in quiet wakefulness than in SWS, consistent with prior observations (Karlsson and Frank, 2009), differences in the prevalence of replay events in quiet wakefulness and SWS are unlikely to underlie the impact of SWS on VTA activity, given that SWS frames with higher spatial content were associated with greater reduction in RR unit activity. These results suggest a functional distinction between brain processes that subserve spatial memory within sessions versus spatial learning across sessions, consistent with prior observations that tie awake hippocampal replay events to within-session performance (Jadhav et al., 2012) yet associate replay events in post-session epochs rich in SWS to cross-session spatial learning (Girardeau et al., 2009; Ego-Stengel and Wilson, 2010).

Memory consolidation in SWS is likely to require broad evaluation of behavioral experiences, and the present results suggest that this evaluation can occur in the absence of their reward prediction contingencies, as represented in the activity of VTA neurons. In this regard, introducing anomalous reward prediction-related activity during sleep via medial forebrain bundle stimulation triggered on place cell activity (de Lavilléon et al., 2015) has been recently demonstrated to drive goal-directed spatial behavior in wakefulness. In neuropsychiatric diseases such as addiction or obsessive compulsive disorders, such anomalous associations could contribute to maladaptive behaviors.

In contrast to the state-dependence of VTA-hippocampal interactions, neurons of the ventral striatum have been found to coordinate with hippocampal replay in SWS (Pennartz, et al., 2004; Lansink et al., 2009). One possible explanation for this distinction, consistent with the suggested role of dopamine as a teaching signal, is that VTA dopamine activity stabilizes and links replayed sequences in quiet wakefulness across brain regions for subsequent consolidative processes in SWS.

It is notable that replay events in these recordings were biased in their spatial content towards reward sites. The basis for this bias remains to be determined and may be driven by a number of factors not examined here, including the presence or expectation of reward, as well as differences in the dwell times, behavioral states, and behavioral repertoires manifested at reward and nonreward locations.

In this study, we observed a preferential engagement of RR units with the reward representation of centrifugal compared to centripetal replayed spatial content, while we did not detect a preference for RR units for the reward representation of reverse compared to forward replayed spatial content. These results are broadly consistent with the previous proposal that dopamine may function to propagate expected value across reactivated hippocampal sequences (Foster and Wilson, 2006).

We also observed greater coordination of RR cells with replayed reward locations in the SWM task compared to the linear track, raising the possibility that VTA-hippocampal coordination at SPW-R events may reflect task contingencies. However, this result should be considered with caution given the limited sample size acquired in each task. Although we did not detect the preferential coordination of RR cells with replayed reward locations immediately after or immediately prior to successful choice behavior, as compared with errors, it remains possible that RR unit coordination with replayed reward locations could reflect (or predict) choice behavior on longer timescales. Of note, the experimental design was not intended to dissect the relationship of other task dependent features, such as uncertainty, to hippocampal-VTA coordination, and this will be worth pursuing in future experiments.

Interestingly, we found clear differences between RR neurons and nonRR neurons in their engagement with the hippocampus. A higher proportion of SPW-R modulated neurons were RR, RR neurons were more biased to fire in relation to replayed reward locations, and RR neurons demonstrated stronger phase-locking to the hippocampal theta rhythm. These results suggest that RR and nonRR neurons represent distinct functional classes of cells, perhaps associated with different cell types (Cohen et al., 2012; Lammel et al., 2008; Margolis et al., 2012; Hnasko et al., 2012), that differentially contribute to hippocampal-dependent spatial memory. Given the uncertainty in the confidence with which dopamine cells can be identified on the basis of electrophysiologic criteria (Ungless and Grace, 2012; Cohen et al., 2012; Margolis et al., 2006), however, we chose not to restrict our analysis to putative dopamine cells. Even so, over 80% of RR neurons had waveform properties that have been associated with dopamine cells, including a wide action potential and firing rates below 10 Hz.

Models of reinforcement learning have suggested distinct contributions of dopamine to certain forms of learning (Walsh and Anderson, 2014; Doll et al., 2012). The specific, wake-associated coordination of RR VTA neurons with hippocampal activity may mediate the capacity for task-associated replay content to predict future paths to goal locations (Pfeiffer and Foster, 2013; de Lavilléon et al., 2015) and may underlie dopamine’s stabilization of hippocampal replay (Mcnamara et al., 2014). These specific VTA-hippocampal interactions are likely to play a critical role in context-dependent reward seeking behavior. In addition, the state-dependent coordination of VTA reinforcement activity with hippocampal spatial replay events directs attention to the differential processing of spatial memory in wakefulness and SWS.

## Materials and methods

All procedures were approved by the Committee on Animal Care of Massachusetts Institute of Technology and followed the ethical guidelines of the US National Institutes of Health.

### Tetrode implantation and recording

**Figure 8.**
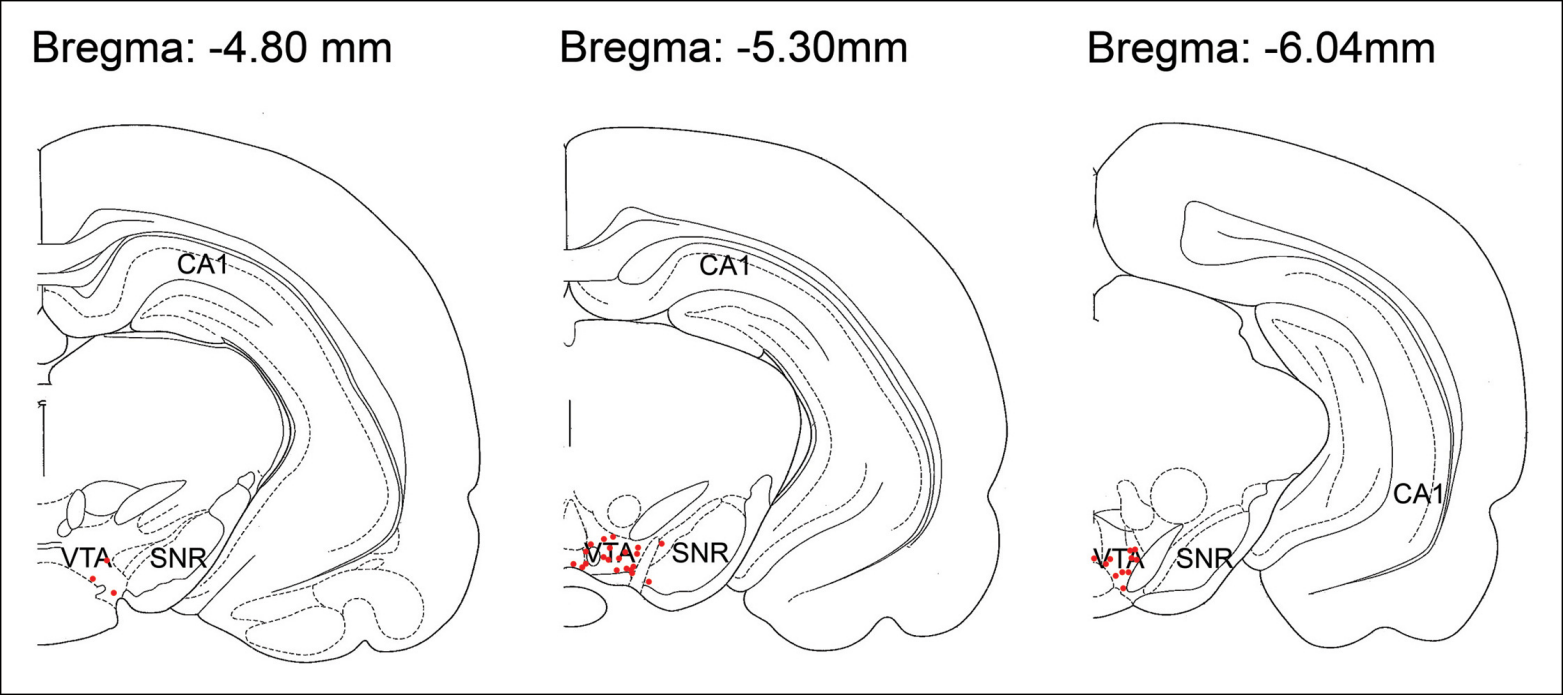
Histological location of tetrode tips targeting the VTA. For each rat, electrolytic lesions marked the tetrode tip locations, and these were mapped onto the stereotaxic atlas of Paxinos and Watson (1998). Tetrode tips under-represent recording locations, which were acquired as electrodes were systematically lowered within the VTA along their tracks. SNR, substantia nigra reticulata. **DOI:** http://dx.doi.org/10.7554/eLife.05360.014

Five male Long-Evans rats (4–6 months old) were implanted under anesthesia (induction: ketamine (50 mg/kg) and xylazine (6 mg/kg); maintenance: isoflurane 0.5–3%,) with 2 arrays of independently movable recording tetrodes (for detailed methods, see Jones and Wilson, 2005). One array of 6–10 tetrodes was directed to the dorsal CA1 pyramidal cell layer (anterior-posterior (AP) -3.6 mm, lateral (L) + 2.4 mm; relative to Bregma). A reference electrode was placed in the white matter above the hippocampal cell layer for differential recordings. An additional array of 8–11 tetrodes was targeted to the VTA (AP -5.3 mm, L + 1.0 mm). A tetrode without unit activity served as the local reference for VTA differential recordings. In one rat, stereotrodes as well as tetrodes were used for VTA recordings. Tetrodes were advanced to their target positions over several weeks. In 4 animals, an additional array of 4–8 electrodes was targeted to the prefrontal cortex for purposes unrelated to the present study. VTA tetrodes were lowered after each recording session and final electrode positions were confirmed with electrolytic lesions and histology (Paxinos and Watson, 1998) after recording was completed (Figure 8).

For hippocampal recording, 1 ms windows around thresholded extracellular action potentials were acquired on-line at 31 kHz, 300-6,000 Hz filtering. In order to retain wave-shape information for VTA units, which often have long waveforms, VTA unit recordings were acquired continuously at 31 kHz, 300–6000 Hz filtering; and subsequently thresholded (60 μV) offline to isolate extracellular action potentials. Local field potentials (2 kHz sampling, 1–475 Hz filtering) were recorded from one electrode on each tetrode.

Head position and direction were monitored using overhead camera tracking of two sets of infrared diodes that were mounted on the headstage and that alternated at 30 Hz each.

### Behavioral training

Animals were trained over 2–4 weeks to run a spatial appetitive choice task (Jones and Wilson, 2005) on an end-to-end T-maze (Figure 1). Each trial consisted of two phases. In the sample phase, rats were directed pseudorandomly to either the left (or right) reward site on the forced side of the maze, where a nosepoke-triggered grain pellet reward could be obtained (MedAssociates (Georgia, VT); Bioserv). In the test phase of the trial, rats traversed the central arm to a choice point, where they chose to go right (or left) in order to win reward on the choice side of the maze. The reward contingency was set up such that if the rat had been forced to turn left in the sample phase, then the correct response in the test phase was to turn right. Individual trajectories between reward sites on forced and choice sides were 300 cm long. After training, the animals were implanted with tetrode arrays. Following recovery from surgery, animals were food deprived to 85% of their free-feeding weights. Animals relearned the task slowly, improving from 60 ± 3% (mean ± s.e.m.) performance in the first three days of behavior to 74 ± 2% in the final three behavioral sessions. Recordings on the spatial alternation task were acquired during the day over 22 days. Two animals ran only on a 200 cm linear track, to acquire food reward at both ends. Sleep sessions were acquired immediately after behavioral sessions in a sleep chamber with opaque walls within the recording room. Animals were housed in individual cages with a 12 hr light-12 hr dark standard light cycle.

### Data analysis

Established software was used for initial identification and characterization of unit activity. This includes identification of well-isolated clusters of spike waveforms and differentiation of putative hippocampal pyramidal cells, hippocampal interneurons, and VTA units (Xclust, M. Wilson). Matlab (MathWorks, Natick, Massachusetts) was used for further data analysis (https://github.com/stephengomperts/eLife_2015). Unless otherwise stated, error bars reflect s.e.m.

Reward responsive (RR) VTA units in the SWM task were identified as those with significantly different firing rates on correct versus error trials during approach to reward (defined as the 2 s window prior to nosepoke) or reward acquisition (defined as the 3 s window following nosepoke; two-sided t-tests, p<0.05 for significance, n = 27). Reward responsiveness on the linear maze was determined by comparing firing rates during reward acquisition to a 3 s window that ended 2 s before nosepoke. These two approaches were highly correlated for the SWM task (r = 0.49, p*=*2.4 × 10^-4^, n = 51). Differential VTA unit activity on correct versus error trials was common in our dataset and is consistent with prior reports in instrumental tasks (Morris et al., 2006; Roesch et al., 2007; Totah et al., 2013). Such results have been interpreted as the representation of choice-associated reward prediction error, formalized for example in the Q-value associated temporal difference prediction error, where Q-value is the value of selecting a particular action at a given state (Morris et al., 2006).

Waveform duration (time from peak to peak) and trough to peak ratio (trough/[peak + trough]) were noted to distinguish two VTA unit populations, as shown previously (Fujisawa and Buzsáki, 2011) (Figure 1C). Waveform duration and the biphasic duration (Ungless and Grace, 2012) were highly correlated (r = 0.79, p=8.53 × 10^-20^, n = 145).

Of 145 VTA units recorded, 84 units (47 RR; 24 nonRR; 13 unclassified due to low firing rate [<0.3Hz]) were recorded concurrent with wake-associated hippocampal activity, over 16 behavioral sessions in 5 rats, with 10 (2, 3, and 5) sessions on the end-to-end T maze; 6 (4 and 2) sessions on the linear track; and in subsequent slow wave sleep. Hippocampal activity in the 5th rat, acquired in 2 sessions on the linear track, was insufficient for position reconstruction and assessment of hippocampal frames, reducing the number of VTA units for replay and frame analyses to 66, acquired over 14 behavioral sessions.

Local field potentials were filtered to obtain hippocampal ripples (Blackman filter; 100–300 Hz) and theta oscillations (4–12 Hz) (Jones and Wilson, 2005). Hippocampal action potentials that exceeded threshold (60 μV) were aggregated as multiunit activity to measure SPW-R-associated bursts in hippocampal activity. Bursts with peak firing rate exceeding 4 standard deviations above the mean, behaviorally constrained to periods of speed < 10 cm/s, were identified as SPW-R multiunit events. The start and end of each event were defined as the times surrounding the event at which the multiunit firing rate fell back to its mean value. Although ripple power was not an explicit constraint for SPW-R multiunit events, 92.8% of SPW-R multiunit events had ripple power exceeding 2 Z scores above baseline (89.0% in replay; 93.5% in nonreplay). The majority of SPW-R events occurred at the force reward sites, where the animals paused longest.

VTA single-unit activity during single trials was summed over repeated trials in a session to generate peri-event time histograms (PETHs) triggered on the start of SPW-R events. The PETH was smoothed with a Gaussian window (σ = 50 ms; similar results were found over a range of σ (30–200 ms). SPW-R modulation amplitude was measured relative to a 300 ms baseline that ended 100 ms before the event, as the baseline-normalized difference between the PETH amplitude measured at the midpoint of the SPW-R event and the mean baseline amplitude. Results were similar using a 5 s baseline ending 1 s before the event. Units with low baseline firing rate (<0.3 Hz) were excluded from analysis to exclude undersampled PETHs. To compute significance of modulation, the SPW-R-aligned raster of each unit was bootstrapped to derive and compare confidence intervals, at the p<0.05 level, of a 50 ms bin at the midpoint of the SPW-R event and the average of the confidence intervals of the 300 ms baseline.

### Position reconstruction

The animal’s location was expressed as a linear distance along the track. For the end-to-end T-maze, the track was linearized by adjoining the 5 segments (Figure 3—figure supplement 1A,B). To deal appropriately with the discrete jumps in the linearized representation, a distance look-up table was constructed for all pairs of densely sampled points along the track. We applied a Bayesian estimation algorithm (Davidson et al., 2009; Zhang et al., 1998) to reconstruct position from hippocampal population activity. We expressed the relationship between neuronal activity and position in an encoding model that incorporated spike waveform amplitude features of unsorted spikes in run epochs with speed > 10 cm/s (‘clusterless decoding’; only putative pyramidal neuron spikes with peak amplitude > 100 μV and spike width > 300 μs were included). For direction reconstruction, we generated an independent encoding model that related running direction in run epochs to spike waveform amplitude features of unsorted spikes. Using the run velocity threshold of 10 cm/s, reward site arrival and departure were well represented. A non-informative uniform prior was used as we did not want to impose any spatial-temporal structure on the estimated positions in SPW-R events.

To verify that position on the track could be accurately estimated from hippocampal population activity, we used a cross-validation procedure by dividing run epochs into alternating 1 s training and testing epochs. The rat’s position (in 10 cm spatial bins) was estimated in non-overlapping 500 ms time bins in the testing epochs and compared to the true location. Decoding performance was assessed by computing the median error and confusion matrices (Figure 3—figure supplement 1C).

We compared the clusterless decoding paradigm to the standard decoding of spike-sorted units. Spatial tuning curves were constructed for all manually sorted hippocampal place cells with peak place field firing rate > 3 Hz. Median error in clusterless position reconstruction was significantly lower than with spike sorting-based reconstruction (clusterless: 8.3 ± 0.5 cm, spike sorting-based 15.2 ± 1.9 cm, p<1.22 × 10^-4^, signed-rank test, n = 14). Mean error in clusterless direction reconstruction was 0.26 ± 0.02.

### Replay-detection

Replay-detection was performed as described previously (Davidson et al., 2009). We applied the clusterless Bayesian estimator to non-overlapping, 25 ms bins during SPW-R events occurring in non-run periods (run speed < 10 cm/s; Figure 3—figure supplement 1D). Excluding running direction, four paths exist on the SWM task that connect the forced choice reward sites to the free choice reward sites (two of which are correct and two incorrect).

For each SPW-R event, a constant speed trajectory was fitted to the sequence of position estimates (Davidson et al., 2009; Kloosterman, 2012) for each of the four possible paths. The best fitting trajectory was selected based on a goodness-of-fit score (‘replay score’), computed as the mean posterior probability within 15 cm of the fitted trajectory. To test if fitted trajectories could be expected by chance alignment of position estimates, the replay score for each candidate event was compared to replay score distributions derived with two shuffles of the data, using the approach described in (Davidson et al., 2009). The first ‘column cycle shuffle’ controls for the linear alignment of consecutive position estimates by circularly shifting the estimated probability distribution over position (PDF) in each candidate event time bin by a random distance. The second ‘pseudo-event shuffle’ controls for bias towards particular locations, by constructing artificial candidate events generated by replacing each PDF in a candidate event with a PDF drawn at random from the complete set of candidate events in each session. Each shuffle was performed 1500 times for each candidate event to obtain sample distributions of the replay score. To increase detection sensitivity of possible replay events on the spatial working memory task, we considered replay events to be those with a Monte Carlo p value<0.05 for both shuffles on at least one path. We considered non-replay enriched events those with a p value>0.2 for both shuffles for all four trajectories. For replay content assessments (below), we used a more stringent criterion for replay detection by performing the shuffles for each putative replay event on the one trajectory with the strongest replay score. We obtained similar results using the standard criterion.

Replay/total (R/T) SPW-R events for each session (s) are as follows: R/T rat 1, s1 173/798; s2 141/434; s3 92/425; s4 71/344; s5 55/203; rat2, s1 120/377; s2 48/210; s3 68/219; rat 3, s1 78/241; s2 30/93; rat 4, s1 65/328; s2 53/275; s3 49/234; s4 64/464; rat 5, s1 -/627; rat 5, s2 -/904.

### Replay content assessment

Distributions of replayed spatial locations were derived by accumulating the spatial posterior probability distribution function across all 25 ms time bins of all replay events, in each recording, for SPW-Rs that occurred while the rat paused at forced reward locations. We determined the temporal delay between hippocampal SPW-R events and VTA activity on the basis of the delay in the SPW-R event-triggered VTA local field potential (84 ± 13 ms). The distribution of VTA spike-associated replay content was derived by accumulating the spatial probability distribution functions for the subset of 25 ms replay time bins that preceded VTA unit spikes by this fixed delay. The probability of reward site content for each recording was measured as the fraction of the distribution of replayed spatial locations that was associated with reward sites. The probability of VTA units coordinating with reward site content (reward site probability) was measured similarly, as the fraction of the distribution of VTA spike-associated replay content that was associated with reward locations. To compare RR and nonRR VTA spike-associated replay content with overall replay content, we computed a reward site bias as follows: Each replay time bin was assigned a 1 (or 0) when the average representation of reward site regions exceeded (or did not exceed) the average representation of nonreward regions. The same binary metric was applied to each RR and nonRR VTA spike-associated replay time bin. From these measures, we computed across the entire dataset the proportion of VTA spike-associated replay time bins that better represented reward site regions compared to nonreward site regions, and we compared this with the proportion of replay time bins that better represented reward site regions, accounting for differences in the number of spike-associated time bins across RR units and nonRR units across recordings, to derive the excess reward site bias. There were 5422 replay time bins, 2269 RR unit spike-associated bins, and 2455 nonRR unit spike-associated bins. On the SWM task, there were 3554 replay time bins and 1046 RR unit spike-associated bins. On the linear track, there were 1868 replay time bins and 818 RR unit spike-associated bins. The reward site bias was highly correlated with the reward site probability (RR units, r = 0.80, p=1.36 × 10^-4^). To explore the sensitivity of the reward site bias to the temporal delay between replayed spatial content and VTA unit activity, we systematically varied the delay in 25 ms steps. Consistent with the latency of the SPW-R-associated VTA potential, the excess reward site bias of RR units was maximal at a 75 ms VTA lag relative to hippocampal activity (data not shown).

To evaluate whether RR unit coordination with replayed reward site representations correlated with choice behavior, we measured the excess reward site bias at force reward site locations immediately after correct and error trials; and separately, immediately prior to correct and error trials. There were 616 RR unit spike-associated bins following correct trials, 243 following error trials, 565 prior to correct trials, and 142 prior to error trials.

For forward and reverse replay content analyses, we measured the reward site bias of replay event time bins that showed strong directional preference for outbound (O) or inbound (I) track direction (direction index >0.5, where the direction index is [O-I]/[O+I]) (Singer et al., 2013). For each replay event, we transformed the direction index of each time bin into an index of forward or reverse content, as follows. Since we restricted our analysis to replay events occurring while the rat paused at force reward sites, for centrifugal sequence replay away from the animal’s location, outbound content reflects forward replay, while inbound content reflects reverse replay. In contrast, for remotely initiated, centripetal replay towards the animal’s location, inbound content reflects forward replay, while outbound content reflects reverse replay. For centrifugal and centripetal replay content analyses, we aggregated forward and reverse replay event time bins together, defining centrifugal replay events as replay trajectories moving away from the current position of the animal and centripetal replay events as replay trajectories approaching the animal. We compared the reward site bias of centrifugal and centripetal replay event time bins and of forward and reverse replay event time bins to the reward site bias of VTA unit spikes occurring with 84 ms delay to those replay time bins. There were 1700 time bins with centrifugal replay content, of which 681 were associated with RR unit spikes and 770 with nonRR unit spikes. There were 1248 time bins with centripetal replay content, of which 585 were associated with RR unit spikes and 602 with nonRR unit spikes. In addition, there were 1561 time bins with forward replay content, of which 699 were associated with RR unit spikes and 720 with nonRR unit spikes. There were 1313 time bins with reverse replay content, of which 567 were associated with RR unit spikes and 652 with nonRR unit spikes.

There were several cases in which we sought to determine whether the excess reward site bias of VTA units compared to hippocampal replay was greater across two comparisons (B vs A compared to C vs A): 1) excess reward site bias of RR units versus nonRR units; 2) excess reward site bias of RR units on the SWM task versus on the linear track; 3) excess reward site bias of RR units after correct trials versus after error trials; 4) excess reward site bias of RR units before correct trials versus before error trials; and 5) excess reward site bias of RR units at forward and reverse replay. For each case, we ran a logistic regression in which we considered each element of the case separately, as well as their interaction. For example, in the first case, we computed a logistic regression to measure the interaction between RR unit-associated reward site bias and replay-associated reward site bias, and nonRR unit-associated reward site bias and replay associated reward site bias. We then compared the interaction term to a distribution of simulated interaction terms assuming the null hypothesis. We considered the reward site bias for replay (A), the reward site bias for RR units (B), and the reward site bias for nonRR units (C). The logistic regression predicted reward site bias as a binary dependent variable (present/absent) with binary predictors of (either B or C = 1 vs A = 0), comparison (B vs A = 1, C vs A = 0), and their interaction, the latter being the predictor pertinent to the research question. Since these hypotheses were in only one direction, we ran 1-tail tests. In order to determine the one tail p value in these nonlinear tests, we performed a nonparametric permutation test of 1,000 iterations of the logistic regression, assuming the null hypothesis (i.e., the coefficient for the interaction is centered at zero). In each iteration, the fraction of reward site bias (A) for each comparison was taken as the overall average of actual estimates separately incremented with a perturbation from a normal distribution. The mean of this distribution was set to 0 and the standard deviation chosen so as to produce simulated interaction regression coefficients with a standard deviation approximately equal to the standard error for the same predictor estimated from the logistic regression of the actual data. The proportion of simulated interaction coefficients greater than or equal to the actual interaction coefficient was taken as the estimated one-tail p value.

To test for bias in the reconstruction algorithm towards reward sites, we first decoded the estimated position of nonreplay events. We did not detect a bias for reward sites in the distribution of spatial locations across nonreplay events (reward site bias 0.424 ± 0.004; probability/spatial bin of content at reward locations 0.032 ± 0.007 bin^-1^; non-reward locations 0.023 ± 0.003 bin^-1^; p=0.1, signed-rank test, n = 14 recordings). Because SPW-R events may encode hippocampal spatial representations other than replay sequences, and because we may have miscategorized some replay events as nonreplay events, we also assessed the output of the reconstruction algorithm in the absence of hippocampal activity. This approach did not detect a preference in the decoder toward reward sites (probability/spatial bin of content at reward locations 0.029 ± 0.006 bin^-1^; non-reward locations 0.023 ± 0.003 bin^-1^; p=0.6, signed-rank test, n = 14 recordings).

### Theta phase analysis

The Hilbert transform of the theta-filtered LFP was used to assess theta phase relationships of VTA units. To evaluate for theta phase preference of VTA units, we computed Rayleigh’s test for uniformity of the circular theta phase distribution of each VTA neuron’s spikes against a unimodal alternative; and we computed the parameters mu and kappa of the von Mises distribution that best fit that distribution (Jones and Wilson, 2005; Siapas et al., 2005), using custom Matlab code. The circular concentration coefficient kappa is inversely related to the variance of the distribution, such that in the limit of kappa = 0, the circular distribution is uniform.

### Frame analysis

Frames were identified as described previously (Ji and Wilson, 2007), within epochs of SWS defined on the basis of low hippocampal theta/delta ratio and clear sleep posture (SWS median duration, range: 1226 s, 793–1998 s). Within SWS epochs, multiunit activity from all tetrodes were combined, counted in 10 ms bins, and smoothed with a Gaussian window, with σ = 30 ms. Frames were identified as periods of high activity between silent periods, with the spike count threshold defined as the spike count at which the spike count distribution reached its first minimum (in 10 ms bins). Clusterless reconstruction was applied to frames of SWS to derive the spatial probability distribution function of each 25 ms time bin within each frame. Spatial content per frame was taken as the average of the maximum decoded probability of each time bin.

## References

[bib1] Cardinal RN, Parkinson JA, Hall J, Everitt BJ (2002). Emotion and motivation: the role of the amygdala, ventral striatum, and prefrontal cortex. Neuroscience and Biobehavioral Reviews.

[bib2] Cohen JY, Haesler S, Vong L, Lowell BB, Uchida N (2012). Neuron-type-specific signals for reward and punishment in the ventral tegmental area. Nature.

[bib3] Davidson TJ, Kloosterman F, Wilson MA (2009). Hippocampal replay of extended experience. Neuron.

[bib4] de Lavilléon G, Lacroix MM, Rondi-Reig L, Benchenane K (2015). Explicit memory creation during sleep demonstrates a causal role of place cells in navigation. Nature Neuroscience.

[bib5] Diba K, Buzsáki György (2007). Forward and reverse hippocampal place-cell sequences during ripples. Nature Neuroscience.

[bib6] Doll BB, Simon DA, Daw ND (2012). The ubiquity of model-based reinforcement learning. Current Opinion in Neurobiology.

[bib7] Ego-Stengel Valérie, Wilson MA (2010). Disruption of ripple-associated hippocampal activity during rest impairs spatial learning in the rat. Hippocampus.

[bib8] Foster DJ, Wilson MA (2006). Reverse replay of behavioural sequences in hippocampal place cells during the awake state. Nature.

[bib9] Fujisawa S, Buzsáki György (2011). A 4 hz oscillation adaptively synchronizes prefrontal, VTA, and hippocampal activities. Neuron.

[bib10] Girardeau G, Benchenane K, Wiener SI, Buzsáki György, Zugaro Michaël B (2009). Selective suppression of hippocampal ripples impairs spatial memory. Nature Neuroscience.

[bib11] Hnasko TS, Hjelmstad GO, Fields HL, Edwards RH (2012). Ventral tegmental area glutamate neurons: electrophysiological properties and projections. The Journal of Neuroscience.

[bib12] Jadhav SP, Kemere C, German PW, Frank LM (2012). Awake hippocampal sharp-wave ripples support spatial memory. Science.

[bib13] Ji D, Wilson MA (2007). Coordinated memory replay in the visual cortex and hippocampus during sleep. Nature Neuroscience.

[bib14] Jones MW, Wilson MA (2005). Theta rhythms coordinate hippocampal-prefrontal interactions in a spatial memory task. PLoS Biology.

[bib15] Karlsson MP, Frank LM (2009). Awake replay of remote experiences in the hippocampus. Nature Neuroscience.

[bib16] Kloosterman F, Layton SP, Chen Z, Wilson MA (2014). Bayesian decoding using unsorted spikes in the rat hippocampus. Journal of Neurophysiology.

[bib17] Kloosterman F (2012). Analysis of Hippocampal Memory Replay Using Neural Population Decoding. Neuronal network analysis.

[bib18] Lammel S, Hetzel A, Häckel O, Jones I, Liss B, Roeper J (2008). Unique properties of mesoprefrontal neurons within a dual mesocorticolimbic dopamine system. Neuron.

[bib19] Lansink CS, Goltstein PM, Lankelma JV, Mcnaughton BL, Pennartz CMA (2009). Hippocampus leads ventral striatum in replay of place-reward information. PLoS Biology.

[bib20] Lee AK, Wilson MA (2002). Memory of sequential experience in the hippocampus during slow wave sleep. Neuron.

[bib21] Margolis EB, Lock H, Hjelmstad GO, Fields HL (2006). The ventral tegmental area revisited: is there an electrophysiological marker for dopaminergic neurons?. The Journal of Physiology.

[bib22] Margolis EB, Toy B, Himmels P, Morales M, Fields HL (2012). Identification of rat ventral tegmental area GABAergic neurons. PloS One.

[bib23] Mcnamara CG, Tejero-Cantero Álvaro, Trouche Stéphanie, Campo-Urriza N, Dupret D (2014). Dopaminergic neurons promote hippocampal reactivation and spatial memory persistence. Nature Neuroscience.

[bib24] Montague PR, Dayan P, Sejnowski TJ (1996). A framework for mesencephalic dopamine systems based on predictive hebbian learning. The Journal of Neuroscience.

[bib25] Morris G, Nevet A, Arkadir D, Vaadia E, Bergman H (2006). Midbrain dopamine neurons encode decisions for future action. Nature Neuroscience.

[bib26] Nádasdy Z, Hirase H, Czurkó A, Csicsvari J, Buzsáki G (1999). Replay and time compression of recurring spike sequences in the hippocampus. The Journal of Neuroscience.

[bib27] O'Keefe J, Dostrovsky J (1971). The hippocampus as a spatial map. preliminary evidence from unit activity in the freely-moving rat. Brain Research.

[bib28] Pavlides C, Winson J (1989). Influences of hippocampal place cell firing in the awake state on the activity of these cells during subsequent sleep episodes. The Journal of Neuroscience.

[bib29] Paxinos G, Watson C (1998). The rat brain in stereotaxic coordinates.

[bib30] Pennartz CMA, Lee E, Verheul J, Lipa P, Barnes CA, Mcnaughton BL (2004). The ventral striatum in off-line processing: ensemble reactivation during sleep and modulation by hippocampal ripples. The Journal of Neuroscience.

[bib31] Pfeiffer BE, Foster DJ (2013). Hippocampal place-cell sequences depict future paths to remembered goals. Nature.

[bib32] Roesch MR, Calu DJ, Schoenbaum G (2007). Dopamine neurons encode the better option in rats deciding between differently delayed or sized rewards. Nature Neuroscience.

[bib33] Schultz W, Apicella P, Scarnati E, Ljungberg T (1992). Neuronal activity in monkey ventral striatum related to the expectation of reward. The Journal of Neuroscience.

[bib34] Schultz W (1998). Predictive reward signal of dopamine neurons. Journal of Neurophysiology.

[bib35] Siapas AG, Lubenov EV, Wilson MA (2005). Prefrontal phase locking to hippocampal theta oscillations. Neuron.

[bib36] Singer AC, Carr MF, Karlsson MP, Frank LM (2013). Hippocampal SWR activity predicts correct decisions during the initial learning of an alternation task. Neuron.

[bib37] Singer AC, Frank LM (2009). Rewarded outcomes enhance reactivation of experience in the hippocampus. Neuron.

[bib38] Totah NKB, Kim Y, Moghaddam B (2013). Distinct prestimulus and poststimulus activation of VTA neurons correlates with stimulus detection. Journal of Neurophysiology.

[bib39] Ungless MA, Grace AA (2012). Are you or aren't you? challenges associated with physiologically identifying dopamine neurons. Trends in Neurosciences.

[bib40] Walsh MM, Anderson JR (2014). Navigating complex decision spaces: problems and paradigms in sequential choice. Psychological Bulletin.

[bib41] Wilson MA, Mcnaughton BL (1994). Reactivation of hippocampal ensemble memories during sleep. Science.

[bib42] Zhang K, Ginzburg I, McNaughton BL, Sejnowski TJ (1998). Interpreting neuronal population activity by reconstruction: unified framework with application to hippocampal place cells. Journal of Neurophysiology.

